# Maternal Larp6 controls oocyte development, chorion formation and elevation

**DOI:** 10.1242/dev.187385

**Published:** 2020-02-26

**Authors:** Hoi Ting A. Hau, Oluwaseun Ogundele, Andrew H. Hibbert, Clinton A. L. Monfries, Katherine Exelby, Natalie J. Wood, Jessica Nevarez-Mejia, M. Alejandra Carbajal, Roland A. Fleck, Maria Dermit, Faraz K. Mardakheh, Victoria C. Williams-Ward, Tapan G. Pipalia, Maria R. Conte, Simon M. Hughes

**Affiliations:** 1 Randall Centre for Cell and Molecular Biophysics, New Hunt's House, Guy's Campus, King's College London, London SE1 1UL, UK; 2 Centre for Ultrastructural Imaging, King's College London, London SE1 1UL, UK; 3 Barts Cancer Institute, Queen Mary University of London, John Vane Science Centre, Charterhouse Square, London EC1M 6BQ, UK

**Keywords:** Zebrafish, Larp6, Knockout, Oocyte, Chorion, Mass spectrometry, Maternal effect

## Abstract

La-related protein 6 (Larp6) is a conserved RNA-binding protein found across eukaryotes that has been suggested to regulate collagen biogenesis, muscle development, ciliogenesis, and various aspects of cell proliferation and migration. Zebrafish have two Larp6 family genes: *larp6a* and *larp6b*. Viable and fertile single and double homozygous *larp6a* and *larp6b* zygotic mutants revealed no defects in muscle structure, and were indistinguishable from heterozygous or wild-type siblings. However, *larp6a* mutant females produced eggs with chorions that failed to elevate fully and were fragile. Eggs from *larp6b* single mutant females showed minor chorion defects, but chorions from eggs laid by *larp6a;larp6b* double mutant females were more defective than those from *larp6a* single mutants. Electron microscopy revealed defective chorionogenesis during oocyte development. Despite this, maternal zygotic single and double mutants were viable and fertile. Mass spectrometry analysis provided a description of chorion protein composition and revealed significant reductions in a subset of zona pellucida and lectin-type proteins between wild-type and mutant chorions that paralleled the severity of the phenotype. We conclude that Larp6 proteins are required for normal oocyte development, chorion formation and egg activation.

## Abstract (precis)

**Summary:** Genome-edited *larp6a* and *larp6b* double-knockout zebrafish appear morphologically and behaviourally wild type and are fertile. Larp6 proteins are, however, required maternally for normal oocyte development and chorion formation and elevation.

## INTRODUCTION

La-related proteins (Larps) are a family of evolutionarily conserved RNA-binding proteins with diverse functions (Maraia et al., 2017). Members of the family have been implicated across a wide spectrum of RNA biology, including tRNA processing, non-coding RNA maturation and metabolism, ribosomal biogenesis through effects on 5′TOP mRNAs encoding ribosomal proteins, and more widely in mRNA translation, in some cases by aiding closed-loop translation (Maraia et al., 2017).

Larp6 is defined by a primary structure, conserved across eukaryotes from plants to people, that includes specific sequences in the RNA-binding region termed La module and a unique C-terminal SUZ-C or LSA motif (Merret et al., 2013). Functional data in vertebrates implicate Larp6 in collagen biogenesis, skeletal muscle formation and cell migration, traits present in metazoa but largely lacking in plants (Glenn et al., 2010; Valavanis et al., 2007; Wang et al., 2009; Zhang and Stefanovic, 2016). Thus, a fundamental and conserved role of the Larp6 sub-family is unclear.

Although La, the founding member of the Larp family, was first discovered as an auto-antigen in individuals with lupus erythromatosis and Sjögren's syndrome, and genetic variation of *LARP1*, *LARP4A*, *LARP4B* and *LARP7* are observed in cancers, the role of *LARP6* in human disease is as yet unclear (Maraia et al., 2017). *LARP6* polymorphism has been linked to susceptibility to type II diabetes, rheumatoid arthritis and coronary artery disease, and the protein is upregulated in some breast cancers and can promote angiogenesis (Assimes et al., 2016; Audo et al., 2015; Shao et al., 2012; Strawbridge et al., 2011). Moreover, *LARP6* expression is altered by anti-hepatocellular carcinoma drug sorafenib and has effects on molecules linked to cell proliferation and migration (Cervello et al., 2012; Weng et al., 2009). In mice, *Larp6* is found in a locus linked to knee injury (Rai et al., 2015). Although none of these medical connections is individually compelling, understanding the key *in vivo* function(s) of Larp6 could shed light on various conditions. However, to our knowledge, no genetic loss of function analysis has been reported in any vertebrate.

Here, we report the generation and characterization of a vertebrate lacking wild-type Larp6 function by genome edited mutation of the zebrafish *larp6a* and *larp6b* genes that reveal a role of Larp6 in chorion formation. When eggs are produced in the ovary, a complex series of layers of extracellular proteins is produced, known as the chorion or vitelline envelope (Selman et al., 1993). The chorion shares many protein components with the mammalian zona pellucida. When the female fish releases eggs, the chorion is tightly apposed to the egg plasma membrane, but the micropyle, a hole through the chorion, allows fertilisation by one sperm. Subsequently, exposure to low-salt solution triggers egg activation, in which rapid fusion of vesicles docked below the plasma membrane aids chorion expansion and stiffening to prevent double fertilisation and form the outermost protection of the developing embryo, as does the mammalian chorion.

We show that single and double homozygous zygotic *larp6* mutants are viable and fertile with functional muscle, skeleton and apparently normal growth. No zygotic behavioural phenotype was observed. However, *larp6a* mutant females produce defective eggs, a phenotype noticeably worsened in eggs from *larp6a;larp6b* double mutant females. The chorion of developing oocytes within the ovary and eggs from mutant females has defective layer structure, reduced elevation, fragility and altered protein composition. Embryos from mutant mothers were misshapen and fragile but, irrespective of zygotic genotype, often developed well within the constricted chorion, hatched and thereafter grew normally.

## RESULTS

### Two Larp6 genes in zebrafish are differentially expressed

The zebrafish genome contains *larp6a* on chromosome 18:173,603-194,844 and *larp6b* on chromosome 11:6,446,235-6,452,492 (GRCz11). The predicted proteins are ∼32% identical, chiefly in the La-motif winged helix-turn-helix domain, RMM1 and LSA regions (Merret et al., 2013). Each zebrafish gene has three coding exons and conserved splice junctions, sequence and synteny with amniotes; *larp6a* shares flanking genes *celf6* (*si:dkey-205h23.2* in zebrafish), *tm2d3* and *tarsl2* (*tars3* in zebrafish) whereas *larp6b* shares *nrtn* and *ranbp3* (*ranbp3a* in zebrafish). In general, Larp6a is more similar than Larp6b to tetrapod Larp6 proteins, with around 50% and 30% identity to human LARP6, respectively (Fig. S1).

**Fig. 1.**
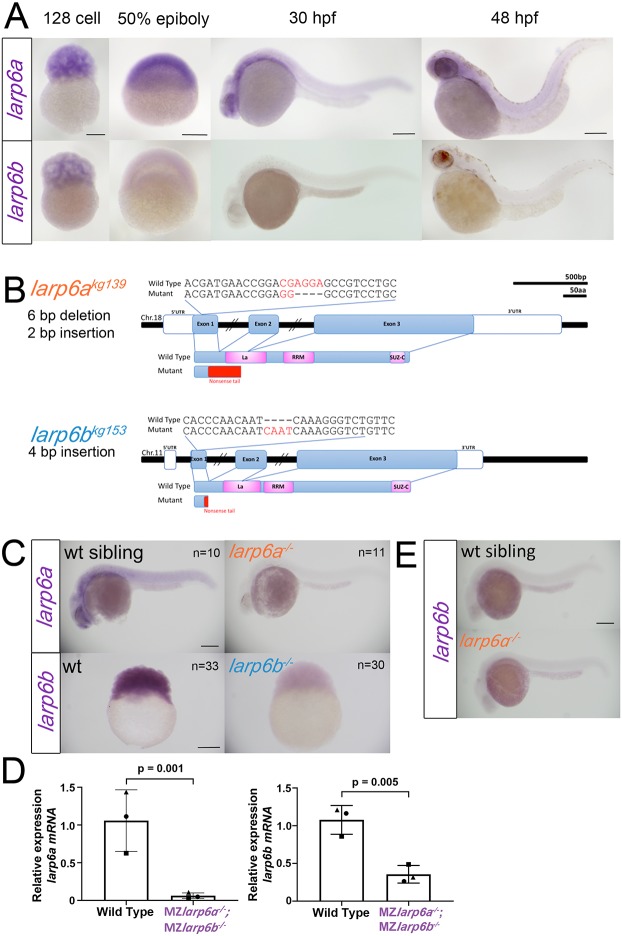
**Genome editing generates likely null alleles of zebrafish *larp6a* and *larp6b*.** (A) *In situ* RNA hybridisation for *larp6a* and *larp6b* at the indicated stages. (B) Schematic of *larp6a* and *larp6b* genes and proteins showing the position of *kg139* and *kg153* mutant alleles. The *larp6a^kg139^* frameshift mutation produces a truncated protein with the first 29 amino acids of Larp6a followed by a 72 amino acid tail lacking both La motif and RRM domains. The *larp6b^kg153^* frameshift allele truncates Larp6b at amino acid 17 with an eight amino acid tail terminating in coding exon 1. There is a lack of in-frame ATG codons near the termination site. (C) *In situ* RNA hybridization for *larp6a* mRNA on genotyped *larp6a^kg139^* mutant and wild-type siblings from a *larp6a^kg139/+^* incross reveals nonsense-mediated mRNA decay (NMD) of mutant *larp6a^kg139^* mRNA at 24 hpf. Eleven out of 47 low expressors were shown to be mutant and 10/47 normal expressors were wild type upon sequence genotyping. As *larp6b* mRNA is primarily maternally expressed, NMD was analysed at the 256-cell stage by *larp6b* mRNA *in situ* RNA hybridization on lays from incrosses of *larp6b^kg153^* F3 mutants or their wild-type siblings (see Table S1). The results shown were observed in 33/33 embryos from a wild-type female and 30/30 from a mutant female. Genotypes of mutant embryos shown were confirmed by sequencing. (D) QRT-PCR on RNA from 1k stage embryos from wild-type or *larp6a^kg139^;larp6b^kg153^* double mutant incrosses confirmed reduction of each mutant mRNA. Symbols indicate results from three individual RNA preparations from three separate pairs of lays. (E) Lack of *larp6b* mRNA upregulation in *larp6a^kg139^* mutants. All images are lateral views with anterior to the left and dorsal upwards, except 128 cell and 50% epiboly, which are animal upward. Scale bars: 200 µm.

The pattern of *larp6a* and *larp6b* mRNA accumulation in embryos was analysed using whole-mount *in situ* hybridisation. *larp6a* mRNA appeared ubiquitous at 128-cell, sphere and 50% epiboly stages, and declined thereafter but remained broadly expressed at 30 and 48 hours post-fertilization (hpf) in the head, trunk and tail (Fig. 1A). In contrast, *larp6b* mRNA was detected in all cells at 128-cell stage, but then declined by sphere stage and became undetectable above background thereafter (Fig. 1A). Thus, both *larp6a* and *larp6b* show expression at early stages, indicating a possible role for Larp6 in early embryonic development, based on maternal loading of mRNA into developing eggs. Widespread *larp6a* mRNA raises the possibility of additional functions for this gene later in development.

### Generation of Larp6 mutant alleles

*Larp6a* was targeted in the first coding exon upstream of the La-motif by CRISPR/Cas9 genome editing and several mutant alleles were isolated, among them *larp6a^kg139^*, which contains a six-base deletion and two-base insertion, leading to a frameshift and stop codon in exon 2 after a 71 amino acid nonsense tail (Fig. 1B). The predicted protein lacks all conserved motifs. Moreover, *in situ* mRNA hybridisation and quantitative PCR revealed that nonsense-mediated decay of *larp6a^kg139^* mRNA, indicating that premature ribosomal termination occurs and therefore that little mutant protein will be produced (Fig. 1C,D). *larp6b* was also targeted in the first coding exon upstream of the La motif by TALEN-based genome editing and the *larp6b^kg153^* allele containing a four-base insertion that predicts a frameshift followed by a stop codon after eight missense amino acids was selected (Fig. 1B). The predicted protein lacks all conserved motifs. Again, nonsense-mediated mRNA decay was detected in *larp6b^kg153^* mutants, suggesting that translation was effectively terminated and little mutant protein would be produced (Fig. 1C). Quantitative PCR confirmed that nonsense-mediated decay reduced maternally derived *larp6a* and *larp6b* mutant mRNAs (Fig. 1D). No upregulation of *larp6b* mRNA was observed in *larp6a^kg139^* mutants, making genetic compensation unlikely (Fig. 1E). Moreover, PCR of RNA isolated from early embryos or ovaries revealed no alternative or aberrant mRNA transcripts (Fig. S2). Thus, it is likely that null alleles for each Larp6 gene were created.

### Larp6a and Larp6b are dispensable for growth and myogenesis

**Fig. 2.**
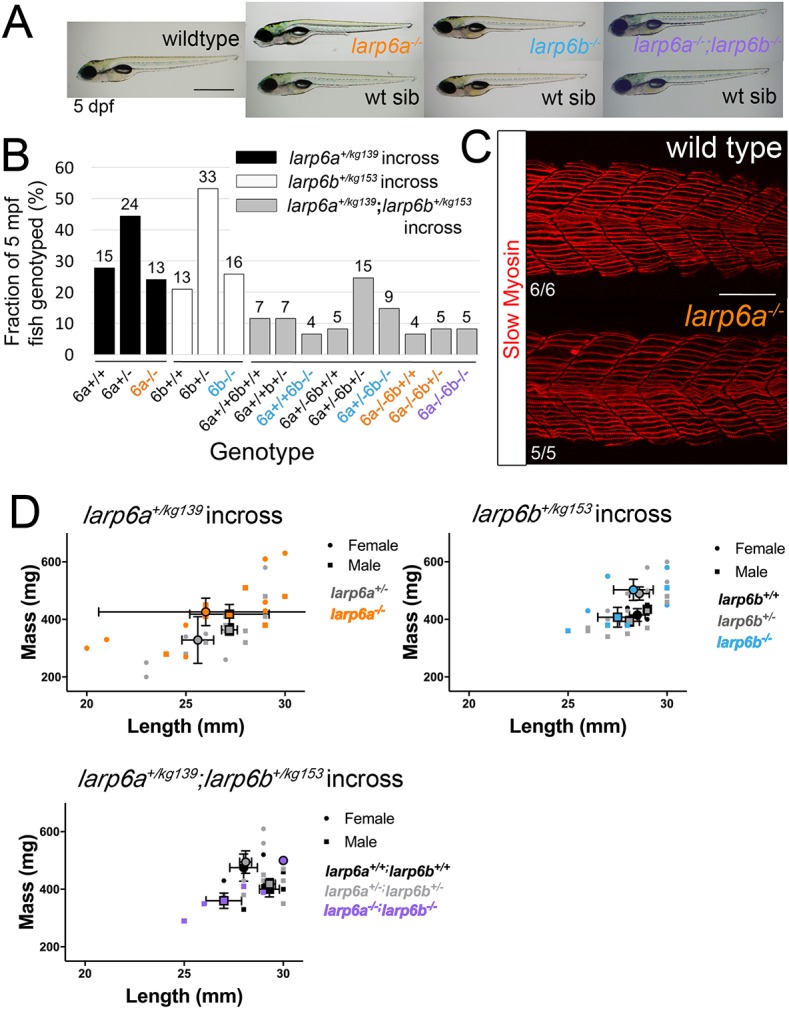
**Zygotic Larp6 mutants appear wild type.** (A) Bright-field images of 5 dpf larvae from wild-type and incrosses of single and double heterozygous carriers. Fish are shown anterior towards the left and dorsal upwards with genotyped mutants above their respective wild-type siblings (wt sib). Inflated swim bladders show that larvae have swimming and swallowing capacity. (B) Adults derived from incrosses of *larp6a^+/^*^−^ (*n*=52), *larp6b^+/^*^−^ (*n*=62) and *larp6a^+/−^;larp6b^+/−^* (*n*=61) fish were co-reared, then genotyped at 5 mpf showing the expected Mendelian ratios. Fish numbers are above each bar. (C) Slow myosin immunofluorescence of 24 hpf embryos. Images of wild type and mutant centred on somite 17/18 reveal no differences in slow muscle fibre formation. (D) Length and weight of sibling individuals from *larp6a^+/−^, larp6b^+/−^* and *larp6a*^+/−^;*larp6b*^+/−^ in-crosses determined at 5 mpf show no significant difference between genotypes. Large symbols reflect means for each sex and genotype±s.e.m. Individuals data points are also plotted. Scale bars: 1 mm in A; 100 µm in C.

Each mutant line was bred to homozygocity at F2, and viable and fertile mutants were obtained at expected Mendelian ratios (Table S1). Previous work in zebrafish suggested that Larp6a is necessary for normal somitic myogenesis (Wang et al., 2009). We examined lays of in-crosses of *larp6a^+/−^* heterozygotes and could discern no motility or somite skeletal muscle defect in mutants. For example, in one cohort of 60 embryos examined using a bright-field microscope at 1, 2, 3, 4 and 5 dpf, while swimming and after anaesthesia, no abnormalities in head, somite, tail, yolk sac, fin, pigmentation or body length were observed. No change in cardiovascular form or function, swimming activity, jaw or pectoral fin movement was detected (Fig. 2A). No significant death was observed in such lays and survival at 5 months of age conformed to Mendelian ratios (Fig. 2B). Slow muscle fibres were analysed in wild-type, *larp6a^−/−^* and sibling *larp6a^+/−^* embryos and larvae, and no defects in slow fibre number, orientation, length, thickness or striation were observed when analysed blind prior to genotyping, or when re-analysed thereafter (Fig. 2C). Growth of all siblings from crossed fish was monitored after rearing in tanks of mixed sex and genotype to ensure competition. No significant difference in length or weight was observed at 5 months between sex-matched wild-type, heterozygote or mutant siblings (Fig. 2D). Subsequent analysis has revealed no preferential loss of mutants compared with their siblings when aged up to 1.5 years. Homozygous *larp6a^−/−^* mutant males and females bred well. We conclude that wild-type *larp6a* function is dispensable for life in our aquarium.

*Larp6b^−/−^* mutants were compared with their siblings in assays similar to those performed on *larp6a^−/−^* mutants. No difference from wild type was observed in myogenesis, survival to adulthood or growth (Fig. 2A,B,D). *Larp6b^−/−^* mutants bred well and have survived as well as their siblings up to 1.5 years of age. Thus, *larp6b* is not an essential gene.

Could *larp6b* function compensate for lack of Larp6a in *larp6a^−/−^* mutants and vice versa for *larp6b^−/−^* mutants? To address this issue definitively, we crossed *larp6b^+/−^* and *larp6a^−/−^* mutants, selected double heterozygotes and then in-crossed these to generate siblings of nine possible genotypes from wild type to *larp6a^−/−^;larp6b^−/−^* double mutants. As with single mutants, double mutants survived and developed normally (Fig. 2A,B, Table S1). Growth of double mutants was indistinguishable from their siblings, when analysed both before and after genotyping (Fig. 2D). We conclude that the zygotic *larp6a* and *larp6b* genes do not functionally compensate for one another during embryonic development.

**Fig. 3.**
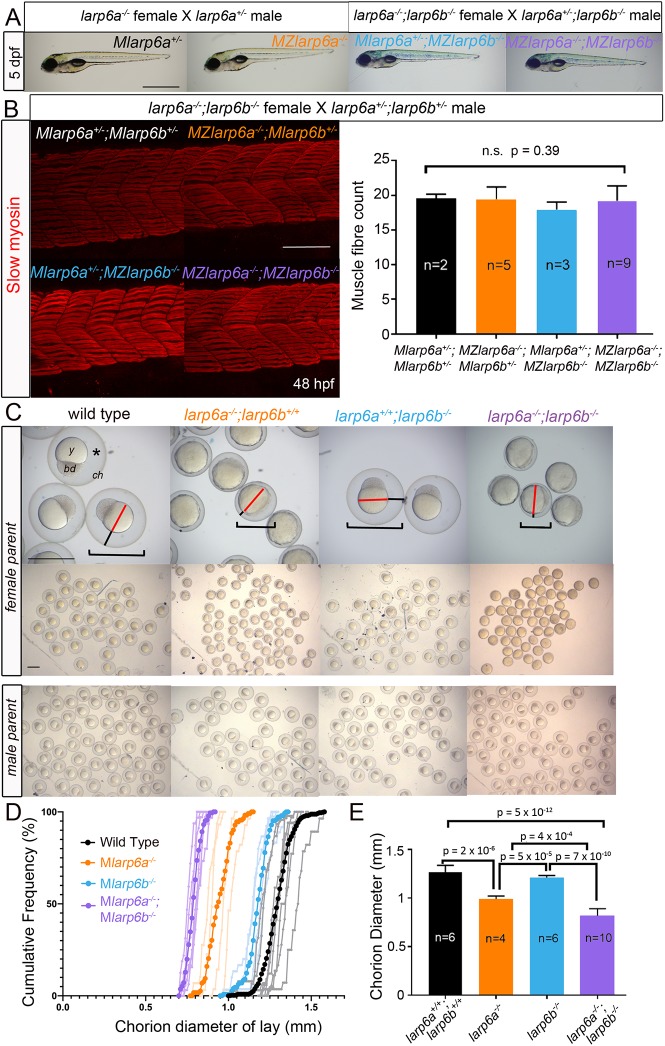
**Maternal effect of *larp6a^−/−^* and *larp6a^−/−^;larp6b^−/−^* on oogenesis.** (A) Bright-field images of 5 dpf larvae from the indicated crosses. Fish are shown with anterior towards the left and dorsal upwards. Inflated swim bladders show that larvae have swimming and swallowing capacity. (B) Slow myosin immunodetection in 48 hpf larvae from a *larp6a^−/−^;larp6b^−/−^* female crossed to a *larp6a^+/−^;larp6b^+/−^* male reveal no differences in slow muscle fibre formation. Images are centred on somite 17/18 and the graph shows the number of slow fibres per myotome±s.d., tested using Welch's ANOVA on SPSS. (C) Bright-field images of lays at 3 hpf from wild-type, *larp6a^−/−^*, *larp6b^−/−^* and *larp6a^−/−^;larp6b^−/−^* females (upper panels) or males (lower panels) crossed to wild-type AB. Embryos from males of any genotype or wild-type females show a fully elevated chorion (ch), translucent and smooth yolk (y), tall and symmetrical blastodisc (bd), large chorion diameter (brackets), and large subchorionic space (asterisk). All embryos from maternal *larp6a^−/−^* mutants have reduced chorion diameter, reduced subchorionic space (black lines) surrounding a yolk cell of unaltered size (red lines) but more opaque and uneven. Lays from *larp6b^−/−^* females are indistinguishable from wild type. Lays from *larp6a^−/−^;larp6b^−/−^* females are like *larp6a^−/−^* embryos, but have even smaller chorion diameter (brackets). (D) Chorion diameter cumulative frequency distribution curves. Light colours indicate individual clutches strong colour indicates the mean for each genotype. (E) Average of the mean chorion diameters of *n* clutches from separate females of indicated maternal genotype±s.d. One-way ANOVA with Tukey's post-hoc test on SPSS. Scale bars: 1 mm in A,C; 100 µm in B.

Given the presence of both *larp6a* and *larp6b* mRNA in the early embryo (Fig. 1A), it is likely that both mRNAs are maternally deposited in the oocyte during oogenesis. Indeed, RNAseq data show that both genes are expressed in the female ovary (Collins et al., 2012). This raises the possibility that one or both wild-type proteins could be present in the egg or translated in the developing embryo. To eliminate this possibility, we analysed lays from females singly mutant for each gene or doubly mutant for both genes, crossed to males of a variety of genotypes, including doubly mutant. In no case was a fraction of embryos observed to have defective myogenesis that correlated with either its own or its mother's genotype (Fig. 3A). Indeed, maternal/zygotic (MZ) double mutant embryos from double mutant females crossed to double heterozygote males survived and underwent myogenesis in the same way as their single mutant or double heterozygote siblings (Fig. 3B). MZ double mutants survive to adulthood and no defects in morphology, growth rate or behaviour were detected. Thus, fish entirely lacking wild-type Larp6 function develop muscle as well as their wild-type siblings.

### Larp6 maternal effect on egg activation and chorion elevation

**Fig. 4.**
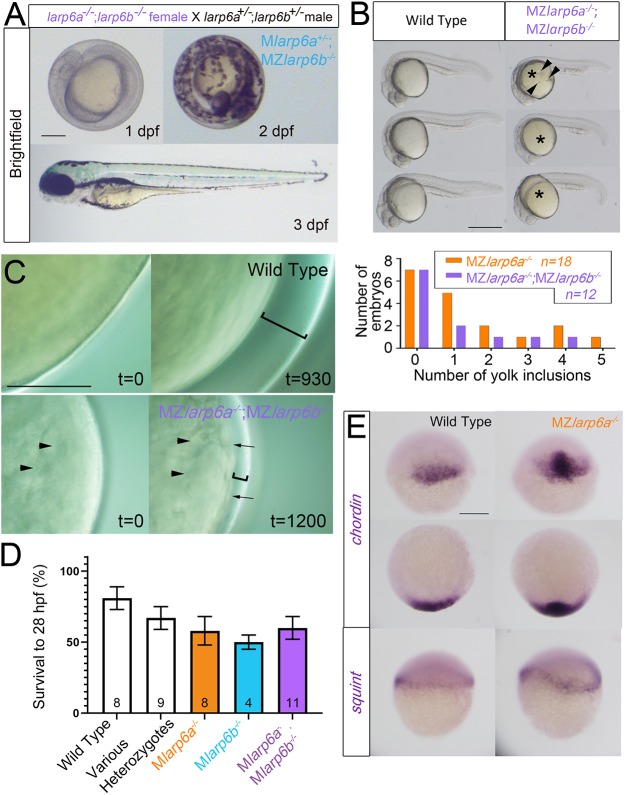
**Maternal-zygotic mutant embryos have defective chorion elevation but normal patterning.** (A) Three successive stages of a single embryo from a *larp6a^−/−^;larp6b^−/−^* female crossed to a *larp6a^+/−^;larp6b^+/−^* male show that embryonic and chorionic defects do not prevent normal hatching and development. The chorion is tight. All zygotic genotypes appeared similar. Scale bar: 200 μm. (B) Yolk inclusions (arrowheads) in 24 hpf larvae of the indicated genotype, quantified in the graph. The yolk (asterisks) is yellow in double mutants. Scale bars: 500 µm. (C) Timelapse imaging of chorion elevation reveals failure in eggs derived from double mutant females. There is reduced chorion elevation (brackets) and uneven cytoplasm in unactivated egg (arrowheads), and uneven egg surface after activation (arrows). Scale bar: 200 μm. (D) Mild reduction in survival of fertilised eggs between 1 and 28 hpf in lays from mutant females. Subsequent survival did not differ between genotypes and no effect of paternal genotype was observed. Data are mean±s.e.m., number of lays is indicated on each column. (E) *In situ* hybridisation for the dorsal marker gene *chordin* and germring marker gene *squint* show that *Mlarp6a^−/−^* embryos are not ventralised compared with wild-type controls at 30% epiboly. Upper panels are lateral views; lower panels are animal views; arrowhead indicates dorsal. Scale bar: 200 µm.

In contrast to the lack of phenotype in mutant zygotes, lack of maternal Larp6 activity led to a defect in newly laid eggs. Immediately after egg-laying in wild-type fish, exposure to low salt/osmolarity water triggers egg activation, during which there is rapid fusion of ‘cortical granule’ intracellular vesicles with the egg plasma membrane, transferring liquid containing salts and proteins from the egg into the sub-chorionic space accompanied by chorion expansion to create a protective ‘egg-shell’ for the developing embryo (Marlow, 2010; Wessel and Wong, 2009). Calculation from the chorion diameter of unactivated and activated eggs reveals that chorion expansion away from the egg/embryo surface is not accounted for by the volume of cortical granules extruded from the activated egg and thus by egg/embryo shrinkage, but represents a genuine increase in volume enclosed by the chorion of about 400%. Eggs laid by *larp6a^−/−^* mutant females (hereafter called *Mlarp6a^+/−^* or *MZlarp6a^−/−^* embryos after fertilization) had small chorions (Fig. 3C-E). Small chorions were observed in 20 lays from six different *larp6a^−/−^* mutant females derived from three separate out-crossed families. *larp6a^−/−^* mutant males (16 lays from five individuals crossed to either wild-type AB or wild-type female siblings) never yielded lays with small chorions. *MZlarp6a^−/−^* embryos had a yellowish/opaque yolk with irregular shape and, as zygotic blastomeres formed and divisions proceeded, the animal region appeared flattened with an irregular surface (Fig. 3C). These data suggested that either egg development or activation was defective. Nevertheless, epiboly and gastrulation proceeded relatively normally and, as described above, embryonic development was normal thereafter (see also below). Maternal genotype was entirely responsible for this phenotype, as neither sibling *larp6a^+/−^* heterozygote (27 lays from nine individuals from two families) nor *larp6a^+/+^* wild-type females laid eggs with chorion defects. Chorions and eggs/embryos derived from *larp6b^−/−^* mutant females (*MZlarp6b^−/−^* embryos) did not show obvious defects (three lays from two individuals) compared with wild-type AB, irrespective of zygotic genotype, although chorion expansion may be slightly reduced (Fig. 3D). Lays from *Mlarp6b^+/−^* heterozygote females appeared wild type (10 lays from four individuals from three families). *MZlarp6a^−/−^;MZlarp6b^−/−^* embryos, however, showed a more severe phenotype (13 lays from ten individuals from two families) than those from *MZlarp6a^−/−^* single mutants, with almost no chorion elevation (Fig. 3C-E and Fig. S3A). Nevertheless, even *MZlarp6a^−/−^;MZlarp6b^−/−^* embryos with tight chorions developed normally and were viable (Fig. 4A). We conclude that Larp6 function is essential for normal chorion elevation, egg activation and morphogenesis of the early embryo.

The chorions of *MZlarp6a^−/−^* or *MZlarp6a^−/−^;MZlarp6b^−/−^* embryos were fragile, such that embryos often broke out of the chorion earlier than normal. Embryonic genotype had no discernible effect on chorion defects. Chorions from mutant mothers were not only less elevated away from the embryo but also tended to stick to surfaces and were more flaccid and more readily torn with forceps, confirming their less robust construction. Lays showed remarkably uniform chorion defects (Fig. 3C-E). Although maternal mutant lays had tight chorions, paternal genotype had no effect (Fig. 3C). The small chorion phenotype was also present in unfertilised eggs from mutant mothers (Fig. 4C). Crosses of *larp6a^−/−^;larp6b^−/−^* double mutant females with wild-type males yielded embryos as defective as double mutant in-crosses, showing that even heterozygous zygotes had defective chorions if derived from a mutant female.

As mutants were generated by genome editing followed by out-crossing to a limited number of wild-type individuals of the AB background, the possibility of selecting for a background mutant allele or an off-target second site mutation was considered. The phenotype is recessive; only mutant females, not their siblings, yield eggs showing the defect. We therefore tested many separate females from multiple generations of in- and out-crosses for the small chorion phenotype (Fig. 3D,E). An average chorion diameter was determined for each lay and compared with embryo/yolk maximal diameter; embryo size was unaltered when chorions were small (Fig. 3E, Fig. S3A). Given the number of crosses we have performed and assuming a single recessive allele in an unlinked gene had been selected in our founder population, we calculate the chance of obtaining the observed correlation with the *larp6a^kg139^* allele as <1:10^10^. Although a second site mutation close to *larp6a* cannot be completely excluded, the similar but milder phenotype in *Mlarp6b*^−/−^ and fully-penetrant exacerbation of the chorion phenotype by removal of *larp6b* function is inconsistent with either a background mutation or an off-target second site mutant. We conclude that Larp6 function during oogenesis and/or egg laying is required for chorion elevation.

### Maternal effect on oocyte development

Despite the tight chorion phenotype and defective blastodisc morphogenesis, most *MZlarp6a^−/−^;MZlarp6b^−/−^* embryos developed normally and were viable (Fig. 4A,D, Fig. S3B). Nevertheless, yolks had altered refractility and irregular shape in *MZlarp6a^−/−^;MZlarp6b^−/−^* lays and some had cytoplasmic inclusions (Fig. 4B). We therefore analysed egg activation by timelapse microscopy to understand how chorion elevation and other aspects of egg activation failed (Fig. 4C). Unactivated eggs from wild-type lays had a smooth surface and bright refraction. Chorions from wild-type lays showed rapid chorion elevation away from the yolk within a few minutes of transfer to low osmotic strength medium (Fig. 4C). In contrast, eggs from double mutant mothers had an altered yolk refractility before activation, irregular yolk surface after activation and failed to show normal chorion elevation (Fig. 4C). Thus, it appeared that eggs from mutants were defective before activation, but also showed a defect in activation.

The *larp6* maternal mutant phenotype shares failure of chorion elevation and yolk inclusions with the *brom bones* maternal effect phenotype previously described, which is due to mutations in another RNA-binding protein hnRNP I (Mei et al., 2009). However, whereas *brom bones* showed a variable penetrance embryo ventralisation phenotype, *MZlarp6a^−/−^* and *MZlarp6a^−/−^;MZlarp6b^−/−^* eggs do not (Figs 2 and 3A). To investigate patterning further, we examined the distribution of *squint* (*ndr1*) and *chordin* mRNAs in developing *MZlarp6a^−/−^* mutants (Fig. 4E). *squint* mRNA is localised around the germring at 30% epiboly in 33/33 wild-type embryos. Unlike *brom bones* (*ptbp1a*) 35/35 *Mlarp6a^kg139^* embryos show no change in level of *squint* mRNA, although slightly less even rings were observed, reflecting the misshapen embryo morphology (Fig. 4E). Similar analysis of *chordin* mRNA, a dorsal marker, showed robust expression in the correct location in all 37/37 embryos at 50% epiboly (Fig. 4E). No axis defects were observed in 41/41 *MZlarp6a^−/−^;MZlarp6b^−/−^* embryos examined in detail. We conclude that loss of Larp6 function yields embryos with activation and chorionic defects, but does not impact dorsoventral axis formation.

### Larp6 function is required during oocyte development for chorion formation

**Fig. 5.**
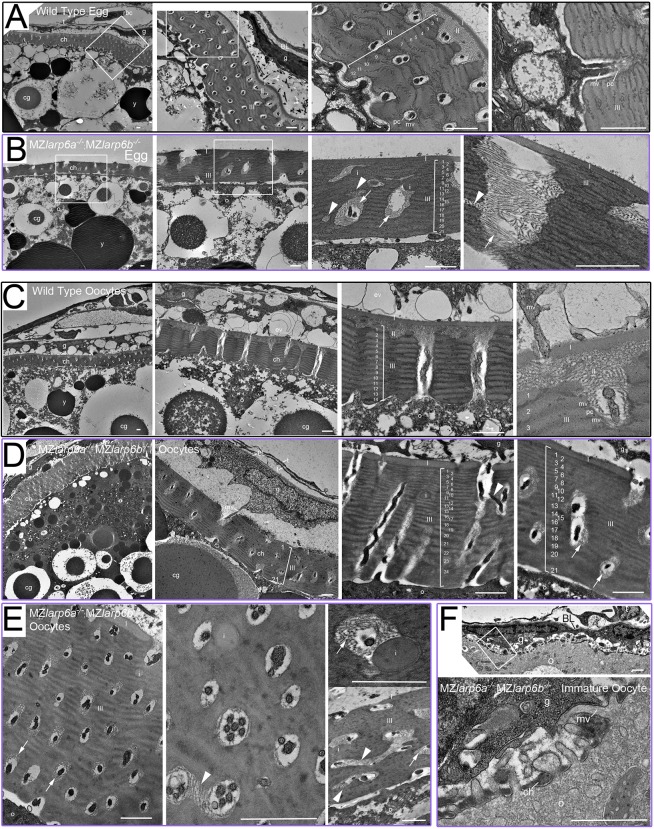
**Larp6 maternal effect on oocyte development and chorion structure.** (A-F) Transmission electron micrographs of mature unactivated eggs (A,B) and ovaries (C-F) from wild-type (A,C) and mutant (B,D-F) *MZlarp6a^−/−^;MZlarp6b^−/−^* mothers. (A) Unactivated wild-type eggs contain yolk platelets (y) and cortical granules (cg), are closely surrounded by a tri-zoned chorion (ch), and are beneath the remains of supporting granulosa (g) and thecal (t) cells separated by basal lamina (BL). There is a smooth thin outer chorionic zone (I), a fibrillar meshwork second zone (II) and a multi-layered thick inner zone (III) consisting of 12-14 alternating dark and light sublayers punctured by regularly spaced transverse pore canals (pc) that sometimes contain microvilli (mv): cellular processes that are ∼100 nm in diameter. (B) Eggs from a mutant mother contain: similar yolk platelets and cortical granules; gross defects in chorionic structure, with irregular pores (arrowheads) and inclusions (i); increased numbers (17-30) of thinner sub-layers in zone III; no zone II; mispositioning of zone II fibrillar material deep into pores (arrows); and an apparently normal zone I. (C) Wild-type ovarian tissue contains eggs showing a series of developmental stages of chorion development. Large oocytes have a 12-14 layered zone III that retain bidirectional processes in regular pores. Zone II fibrillar material is not present deep within the pores. (D,E) Ovarian tissue from a *larp6a^−/−^;larp6b^−/−^* mutant female shows defective chorion structure in several separate oocytes (D) and, at high magnification, mutant oocytes have irregular and branching pores (arrowheads), up to six process profiles per pore canal, disorganised sub-layering in zone III (numbers), absence of a uniform zone II layer, parallel bundling of zone II fibrils and penetration of fibrillar material through the entire depth of pores (arrows) (E). (F) An immature double mutant oocyte lacking yolk platelets and cortical granules had granulosa cells closely opposed to the oocyte plasma membrane with some regions of interdigitating processes initiating chorionogenesis that were indistinguishable from wild type. Boxes show successively magnified areas in A,B (first three panels only) and F. Scale bars: 1 μm.

To determine whether the maternal Larp6 chorion phenotype is apparent in egg development or solely after egg activation, we turned to electron microscopy. Comparison of unactivated *MZlarp6a^−/−^;MZlarp6b^−/−^* or wild-type eggs obtained by squeezing mothers revealed dramatic chorion defects. Consistent with previous studies (Selman et al., 1993), wild-type chorions in eggs from two separate females had a three zone structure, with an outer ∼200 nm uniform zone I lying beneath the granulosa cells, a subjacent fibrillar zone II of variable thickness and a ∼2.5 μm zone III comprising a series of 12-14 regular sublayers, which apposes the oocyte plasma membrane. All three zones were penetrated by regularly spaced pore canals that, when cut transversely, contain one or two microvilli, cellular protrusions about 100 nm in diameter originating from either the oocyte or granulosa cells (Fig. 5A). Chorions of eggs from a double mutant mother showed multiple defects (Fig. 5B). Zone I appeared similar to wild type. Zone II was generally absent, with zone I immediately apposed to zone III. Zone III had more sublayers (18-30) of variable depth and occasional round inclusions reminiscent of zone I material (Fig. 5B). Pores were irregularly spaced, mis-orientated and sometimes branched, which was not observed in wild-type chorions. Strikingly, fibrillar material similar to that in wild-type zone II was observed throughout the depth of pores in mutants, whereas in wild-type chorions it is restricted to the outer sublayers of zone III. Moreover, whereas the fibrils of zone II formed an intersecting meshwork in wild-type chorions, they often formed parallel arrays in mutants (Fig. 5B). Thus, absence of maternal Larp6 function leads to defective chorionogenesis in unactivated eggs.

Examination of internal structure of oocytes from the mutant female did not reveal obvious defects. Cortical granules and yolk platelets were present and appeared normally positioned. Sub-oolemal cytoplasmic structure appeared unaltered and the frequency, size and structure of protrusions penetrating the chorion was similar to wild type. Granulosa and thecal cell layers also showed no obvious differences between eggs from the mutant and wild type. Nevertheless, some individual pores contained up to six cellular protrusion profiles, a phenomenon not observed in wild type (Fig. 5B). Pores containing more protrusion profiles were not uniformly or randomly distributed, but tended to cluster in regions of a single mutant chorion. These changes in cellular structures present prior to egg activation may contribute to the subsequent chorion phenotype.

To assess when during egg development chorion formation becomes defective, ovaries were subjected to electron microscopy after egg extrusion by squeezing, permitting analysis of oocytes of varying maturity (Fig. 5C,D and Fig. S4). Wild-type ovaries contained oocytes at many stages of development (Fig. S4). We identified immature oocytes based on small size and lack of yolk platelets or cortical granules, and observed that chorion formation commenced with a layer of dense extracellular material appearing between protrusions on the surface of the oocyte. This material had some similarity to zone I of later chorions (Fig. 5F). At such stages mutant oocytes appeared similar to wild type. Wild-type oocytes from stage II onwards had a three zone chorion structure (Selman et al., 1993), but zone III initially contained fewer sub-layers than the 12-14 present in mature eggs. Zone II was already composed of fibrils that separated zones I and III, and extended only a short distance into pores, never being observed in the inner half of zone III (Fig. 5C). In contrast, mutant ovaries were defective from as soon as a three-zone structure was apparent. Zone II fibrillar material extended deep into pores and generally failed to separate zones I and III. Pores were misoriented and branching, and some contained more cell protrusions (up to five were observed; Fig. 5D). Oval inclusions of material reminiscent of that in zone I were observed adjacent to pore canals and deeper in zone III (Fig. 5D,E). We conclude that double mutant females produce oocytes with a defective vitelline envelope from early in chorionogenesis.

### Defective chorion composition in eggs from mutant females

To gain insight into the *MZlarp6a^−/−^;MZlarp6b^−/−^* phenotype, we first analysed the protein content and processing of the chorion (Fig. S5). There were several abundant polypeptides in wild-type chorions with characteristic sizes, most of which were still present in *Mlarp6a^−/−^* chorions (Fig. S5). A few proteins, however, showed distinct band intensities and positions in *Mlarp6a^−/−^* chorions, with appearance of bands around 90 and 40-50 kDa (Fig. S5). *Mlarp6b^−/−^* chorions showed similar changes but to a lesser extent. Chorions from eggs laid by *larp6a^−/−^;larp6b^−/−^* females had a similar band pattern but reproducibly stained more weakly with Coomassie Blue (Fig. S5). Nevertheless, and despite the fragile nature of *Mlarp6a^−/−^* and *Mlarp6a^−/−^;Mlarp6b^−/−^* chorions, Bradford and A_280_ assays showed that total extracted protein per chorion did not differ significantly between wild type and each of the three mutants. We conclude that loss of either Larp6a or Larp6b alone, or both Larp6 proteins during oogenesis leads to numerous changes in chorion proteins, possibly reflecting altered processing.

**Fig. 6.**
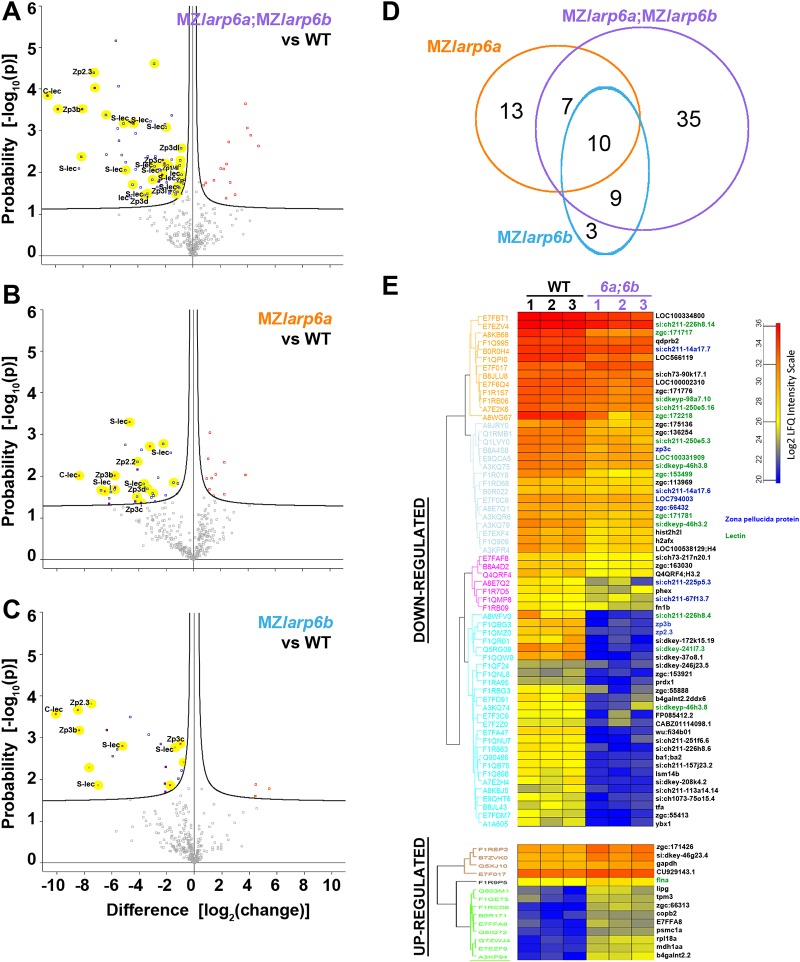
**MS/MS analysis of Larp6 mutant zebrafish chorions reveals altered composition.** (A-C) Volcano plots showing the extent and significance of altered proteins in samples of 30 chorions from MZ*larp6a*;MZ*larp6b* (A), MZ*larp6a* (B) and MZ*larp6b* (C) incrosses. (D) Venn diagram showing overlap of significantly downregulated chorion proteins in each mutant. (E) Hierarchical clustering of the protein intensity values for significantly altered proteins across the triplicate wild-type and double mutant samples. Uniprot ID is on the left. Gene IDs (where available) are on the right: green, lectin proteins; blue, zona pellucida proteins.

Next, we employed liquid chromatography coupled by tandem mass spectrometry (LC-MS/MS) to characterise the protein content of chorions from independent triplicate lays of wild-type or of mutant females crossed with mutant males to create maternal zygotic (MZ) mutant lays (Fig. 6 and Table S2). The strongest protein intensities in wild-type chorion preparations included 14 zona pellucida proteins and other secreted proteins associated with oocytes (Table S2). Many have no defined function, but analysis using GeneTree (ensembl.org) revealed homology with large gene families present across the vertebrates (Table S2) (Liu et al., 2006). Protein families represented more than once included Zp3, Zp2, Adgrg, Vtg*,* SUEL-type and C-type lectins, Oosp3, mucins and Mfap4. The predominance of the expected kinds of proteins in multiple samples provided confidence that the analysis was accurate and reproducible.

To ensure detection of many proteins in each sample irrespective of genotype, we ran equal quantities of total protein from each sample, which means that our analyses compare relative levels of different proteins, not changes in absolute amount of protein per chorion. Altered proteins were identified on the basis of fold-change and *P*-value, derived from *t*-test analysis (Fig. 6A-C). In the triplicate MZ experiment, 17% (61/360) of identified proteins were significantly decreased (*P*<0.05) in *MZlarp6a;MZlarp6b* lays compared with wild type, and these included nine zona pellicuda (zp) proteins, 15 lectins, a Mfap4 protein and an Oosp3 protein (Fig. 6A,D,E). Some protein intensities were more than three orders of magnitude lower in *MZlarp6a;MZlarp6b* mutant chorions compared with wild type, indicating essentially complete loss; most of these were lectins or Zp proteins (Fig. 6A,E, top of cyan group). Most (67%) of significantly decreased proteins encode known extracellular matrix and/or predicted secreted proteins, consistent with a specific chorion defect (Table S2). Some constitute novel chorion protein gene families; e.g. the highly-downregulated FLO11-like mucin glycoprotein encoded by *si:dkey-172k15.19* is a member of a unique zebrafish Gene Tree group that contains tandemly duplicated genes on loci in chromosomes 4 and 10, four of which were detected in the LC-MS/MS analysis of wild-type chorion (Table S2). Moreover, 56% of decreased proteins are encoded by genes that are most abundantly expressed in female gonad or mature ovarian follicles (uniprot.org). Of 17 detected zp proteins encoded by distinct genes, a subset of nine showed significantly reduced MSMS intensity, whereas the remaining eight were not significantly changed. The large drops in intensity of subsets of Zp proteins and various lectins strongly suggest their incorporation into chorion is profoundly altered.

The extent of change in MS/MS data in the single MZ mutants matched the severity of their phenotypes. *MZlarp6a* chorions showed fewer changes than *MZlarp6a;MZlarp6b* mutants (Fig. 6B,D). *MZlarp6a* chorions had 9% (30/338) of protein intensities significantly downregulated, a majority of which were also significantly reduced in *MZlarp6a;MZlarp6b* mutants. Strikingly, despite the lack of a visible chorion elevation phenotype in *MZlarp6b* lays, 6% (22/353) of detected protein intensities were significantly downregulated (Fig. 6C,D). Whereas 86% (19/22) of downregulated protein intensities in *MZlarp6b* chorions were also downregulated in *MZlarp6a;MZlarp6b* chorions, half (9/19) were not significantly altered in *MZlarp6a* chorions (Fig. 6C; Table S2). These findings suggest that Larp6b performs unique functions in chorion formation that are not shared with Larp6a and explain the stronger phenotype of double mutants.

We also detected significant increases in a smaller number of protein intensities in single and double mutant lays (Fig. 6E and Table S2). In double mutant chorions, only 14 protein intensities were increased, nine of which were among the least abundant in wild-type chorions, and therefore likely reflect a relative increase due to the reduction of abundant chorion proteins (Fig. 6E). Although further analysis will be needed to understand how loss of Larp6 function causes these differences during chorion formation, we conclude that Larp6s are required during oogenesis to ensure correct protein composition of the chorion.

## DISCUSSION

The data presented allow three major conclusions. First, that Larp6 proteins are not essential for life; simultaneous MZ mutation of both *larp6a* and *larp6b* genes yields viable, fertile zebrafish with normal development and growth, and adult form and behaviour. Second, that a number of proposed functions for Larp6 proteins do not receive support from genetic ablation of Larp6 function. Third, that Larp6 has a function during egg chorion development, which may indicate an evolutionarily conserved function in gametogenesis.

### Muscle differentiation without Larp6

Based on gain- and loss-of-function experiments in zebrafish, Larp6a was proposed as a regulator of myogenesis (Wang et al., 2009). Our data do not support this view. Our mutants are likely nulls because: (1) the N-terminal location of our mutations predicts loss of all known Larp6 functional protein domains; (2) the strong nonsense-mediated mRNA decay in our mutants shows that ribosomes are unable to progress along the mRNA and therefore that little truncated and no full-length protein will be produced; (3) no altered splicing or promoter use was observed; and (4) the presence of maternal effect phenotypes indicate that the mutant proteins, if produced, are indeed defective. There is debate in the research community concerning the possibility of genetic compensation in null mutants when partial loss of function, which is induced by antisense approaches, yields dramatic phenotypes (El-Brolosy et al., 2019; Ma et al., 2019; Stainier et al., 2017). Although such effects could reconcile our findings with those of Wang et al. (2009), as we have mutated all alleles of *larp6a* and *larp6b* genes, thereby preventing the most obvious methods of nonsense-induced transcriptional compensation, we prefer the simpler interpretation that artefacts associated with morpholino off-target effects, perhaps combined with true loss of target gene function, sensitise certain tissues to morpholino toxicity. We have previously described an instance of morpholinos yielding off-target phenotypes restricted to the expressing tissue in cardiomyogenesis (Hinits et al., 2012). Until a molecular mechanism for any compensation is demonstrated, we believe the parsimonious conclusion is that endogenous Larp6 protein has no major function in zebrafish myogenesis. Whether Larp6a overexpression can enhance muscle growth, as reported by Wang et al. (2009), and whether a more subtle defect in adult muscle might be present in mutants has not been addressed by our study and remain interesting possibilities.

### Larp6 is not required for normal type 1 collagen function

Larp6 has been shown to bind to evolutionarily conserved elements in certain collagen mRNAs (Cai et al., 2010; Martino et al., 2015). Moreover, siRNA knockdown of LARP6 in human fibroblasts reduced accumulation of collagen α1(I) and α1(2) polypeptides (Wang and Stefanovic, 2014). Type 1 collagen is abundant, forms fibrils and is mainly distributed in bone, dermis, tendon, ligament and cornea (Gelse et al., 2003). In zebrafish, three type 1 collagen genes exist, *col1a1a*, *col1a1b* and *col1a2*, which code for α1(I), α3(I) and α2(I) chains, respectively, and all retain the conserved Larp6-binding element in their 5′ UTR (Cai et al., 2010; Castro et al., 2017). The three mRNAs are expressed in epidermis, fin, muscles, osteoblasts and fibroblast of the tendons and ligaments (Gistelinck et al., 2016; Thisse and Thisse, 2004). A recent knockdown study has suggested that *larp6a* morphants show defective epidermal wound repair (LeBert et al., 2018). If Larp6 proteins regulate type 1 collagen production in zebrafish, *larp6* mutants might have severe phenotypes similar to type 1 collagen subunit mutations that produce the various bone dysplasias, such as osteogenesis imperfecta (Asharani et al., 2012; Fisher et al., 2003; Van Eeden et al., 1996). In zebrafish, missense mutation in *col1a1a^+/dc241^* causes dominant defective fin growth, whereas *col1a1^dc241^* homozygotes fail to develop swim bladders or to feed (Fisher et al., 2003). Mutations in other type 1 collagen subunits yield similar phenotypes (Henke et al., 2017). Our mutants contained no obvious bone or fin defects, and thus yield no indication that Larp6 is required for type 1 collagen formation in zebrafish. As most pathogenic mutations affecting type 1 collagen are dominant, however, Larp6 could nevertheless play a role in collagen metabolism, as previously suggested (Parsons et al., 2011).

Only animals have collagen, yet Larp6 genes exist throughout eukaryotes (Martino et al., 2015; Merret et al., 2013). Recent data raise the possibility of broader function for Larp6. In *Xenopus*, a morpholino loss of function study has implicated LARP6 in ciliogenesis, a process occurring widely across eukaryotes (Manojlovic et al., 2017). However, our MZlarp6 double mutants show none of the strong defects reported in zebrafish ciliogenesis mutants (Zhao and Malicki, 2007). Nevertheless, as Larp6 can also bind to conserved microRNA targets (Treiber et al., 2017), it is likely that Larp6 function involves interaction with a range of RNA targets and these might regulate different RNAs across species and life stages.

### Larp6 function in egg development

We find that Larp6 function is important maternally for proper egg development. Both *larp6a* and *larp6b* are expressed in developing oocytes, as reflected by the presence of their mRNAs in newly laid eggs, so it is possible that the Larp6 requirement is cell-autonomous to the developing oocyte. Salmon *larp6b* is also expressed in oocytes in the ovary (Kleppe et al., 2017). The maternal requirement for Larp6a and Larp6b proteins that our mutants reveal indicates that several aspects of egg development are Larp6 dependent. Most noticeably, chorionogenesis and elevation upon egg laying are defective, defects similar to that reported in the unmapped *claustro* mutant (Pelegri et al., 2004). The defect reflects changes in the chorionic material surrounding oocytes prior to laying. There could also be defects in the sub-chorionic components secreted into the chorionic space from cortical granules upon egg activation (Becker and Hart, 1999; Marlow, 2010; Wessel and Wong, 2009), although we observed no defect in cortical granule ultrastructure. Our data show that chorions are physically different from wild type from early during their formation and, when isolated 3 h post-laying, have differences in protein content, reflecting the sum of altered assembly during egg development and, possibly, distinct processing/retention during chorion elevation.

*Mlarp6a^−/−^* and double mutants have similarities to and differences from the *brom bones hnRNP1* mutant (Mei et al., 2009). The reduced chorion elevation and strength, misshapen embryo and visible yolk inclusions are similar. These similarities suggest the proteins may work in a common pathway. The positioning and translational regulation of RNAs in the egg has long been known to provide information for embryo patterning in a variety of animal species (Pushpa et al., 2017; Rojas-Ríos and Simonelig, 2018). However, whereas maternal *brom bones* mutants have a partially penetrant ventralised phenotype (Mei et al., 2009), no such defect was observed in single *Mlarp6a^−/−^* or double *Mlarp6a^−/−^;Mlarp6b^−/−^* mutants, suggesting that Larp6 does not provide positional information in the embryo. These differences suggest Larp6 and hnRNP1 proteins may work in parallel pathways, that each contribute to efficient egg activation.

*brom bones* mutants are thought to affect primarily egg activation (Mei et al., 2009). In contrast, our data clearly reveal defects in Larp6 mutant chorionogenesis within the ovary. It is striking that the poorly described process of chorionogenesis involves production of a complex extracellular matrix structure in association with microvilli, fine cell protrusions extending from both the developing oocyte and its surrounding granulosa cells. Our recent work has implicated Larp6 in mRNA localisation and translation in cellular protrusions (Dermit et al., 2019 preprint). We speculate, therefore, that altered mRNA metabolism within microvilli might contribute to the observed defects in chorionogenesis in Larp6 mutants.

Upon egg activation, the extracellular chorion approximately doubles its surface area in a few minutes, implying that energy stored within the unexpanded chorion drives chorion expansion once egg activation releases a latch. The mechanisms underlying assembly and expansion of this molecular machine are mysterious, but activation involves release of enzymes from cortical granules that may open the latch (Becker and Hart, 1999; Marlow, 2010; Wessel and Wong, 2009). The observed defects in mutant chorion structure may prevent energy storage or latch release. Alternatively, microvillar retraction from pores prior to egg laying (Le Menn et al., 2007; Selman et al., 1993) could be defective in eggs from mutant females. Our observation of fine strands of material linking processes to chorion zone III in unactivated eggs and the penetration of zone II fibrils into pores in eggs from mutant females suggest mechanisms by which egg activation could fail to release the latch of chorion elevation in mutants. Further structural analysis may shed light on precisely how chorion expansion fails in eggs from mutant females.

MS/MS analysis of zebrafish chorions yields preliminary insight into chorion composition. Among over 300 detected proteins, we observed numerous zona pellucida proteins, lectins, mucins, matrix metabolising enzymes and other secreted or extracellular matrix proteins. A number of known or predicted intracellular proteins, including histones, ribosomal proteins and vitellogenins (which are transported through the follicular layer and stockpiled in egg cytoplasm) were also detected; such proteins could reflect contaminants from embryonic material or follicular cells that remained attached to the chorion. Our methods do not permit quantitative comparison of absolute abundances between distinct proteins, as MS peak intensities are differentially influenced by peptide properties. Nevertheless, our data on zebrafish chorion protein composition should facilitate understanding of the formation of this spectacular acellular molecular machine (Shibata et al., 2012).

Paralleling their reduced strength and elevation, chorions from mutant mothers show dramatic reductions in MS protein intensities for subsets of chorionic proteins. Double mutants showed the most numerous significant changes, whereas single MZ*larp6a* mutants showed fewer and MZ*larp6b* mutants the fewest changes, but in primarily overlapping proteins. These findings indicate that loss of Larp6 function causes specific change in chorion composition, not simply reduction in chorion mass. Indeed, total extracted protein was similar, irrespective of maternal genotype. We speculate that Larp6 functions to regulate translation or localization of a subset of mRNAs required for chorion assembly. Specific mRNA targets remain to be determined, but we note that no change in collagen proteins was observed. Indeed, no collagen was identified in the chorion samples. Instead, specific chorion proteins showed differing degrees of reduction/loss. Larp6 has previously been suggested to bind to specific stem loop structures in collagen and other RNAs (Stefanovic et al., 2014; Treiber et al., 2017); analysis of sequence and predicted secondary structure of 5′UTRs of mRNAs encoding the significantly downregulated proteins failed to find any common motifs that would suggest Larp6-binding sites. The strong reduction in a specific subset of zona pellucida and carbohydrate-binding lectin proteins nevertheless suggest a route to understanding the role of Larp6 role in chorionogenesis, which must explain the altered pore structure and increase in number of sublayers in chorion zone III.

Our genetic analysis of Larp6 function in an animal make a striking contrast with parallel analysis in the plant *A. thaliana*, which has three Larp6 genes. T-DNA-mediated insertional mutation of *larp6c*, a gene expressed primarily in pollen (the male gamete), led to viable plants with diminished male fertility due to a partially penetrant defect in pollen tube guidance accompanied by widespread mRNA changes in pollen (M.R.C. and Cecile Bousquet Antonelli, personal communication). Plant LARP6C contains an additional sequence motif capable of interacting with poly(A)-binding protein that is not present in the more widely expressed LARP6A (Merret et al., 2013). It is unclear, therefore, whether plant LARP6C has acquired novel functions compared with both LARP6A and animal Larp6 proteins. Whatever the case, the finding that Larp6 is involved in gametogenesis across eukaryotes suggests that regulation of RNA localisation or metabolism in gametes may be a conserved role of the Larp6 family.

### Conclusions

Like other Larp proteins, Larp6 RNA-binding proteins have been broadly conserved across eukaryotes (Martino et al., 2015; Merret et al., 2013). They must, therefore, play a fundamental role in cells with a cytoskeleton, endomembrane systems and linear chromosomes replicated by mitosis. Nevertheless, Larp6 orthologues in *S. cerevisiae* and *C. elegans* appear to have been lost. Our finding that zebrafish Larp6 is dispensable for life in partially inbred fish in the aquarium environment leaves the deep conserved function unknown, but suggests it may be required for successful eukaryotic response to vicissitudes of life. Among such vicissitudes is the trauma to newly-laid zebrafish eggs while settling into silt and during the subsequent 2 days before hatching, when predation may be severe (Engeszer et al., 2007; Spence et al., 2008). The lack of an expanded and robust chorion in embryos lacking Larp6 would likely greatly reduce survival in the wild.

## MATERIALS AND METHODS

### Zebrafish lines and maintenance

*Danio rerio* lines used were reared at King's College London on a 14/10 h light/ dark cycle at 28.5°C with adults kept at 26.5°C, with staging and husbandry as described previously (Westerfield, 2000). Embryos/larvae were reared at 28.5°C in the dark, except for periods outside the incubator. *larp6a^kg139^* and *larp6b^kg153^* mutant alleles on AB background were genotyped by high-resolution melt analysis (HRMA), followed by sequencing using primers indicated (Table S3). Briefly, HRM primers amplified DNA fragments of 102 bp, 106 bp, 115 bp and 111 bp, and sequencing primers amplified DNA fragments of 317 bp, 321 bp, 280 bp and 276 bp *larp6a^+/+^*, *larp6a^kg139^*, *larp6b^+/+^* and *larp6b^kg153^* alleles, respectively. All experiments were performed on zebrafish derived from F2 or later generation, in accordance with licences held under the UK Animals (Scientific Procedures) Act 1986 and later modifications and conforming to all relevant guidelines and regulations.

### Generation of larp6 mutants

Genome editing was adapted from Hwang et al. (2013). REAL assembly created TALENS targeting *larp6b* in exon 1: forward 5′-TCTTCCATTGTGAAGACA-3′ and reverse 5′-TCTGTTCTCATACACTGA-3′. CRISPR target site 5′-GGAGGACGATGAACCGGACG-3′ in the first coding exon of *larp6a* were selected using ZiFiT (Sander et al., 2010) and potential off-targets minimised using the specific ZiFiT tool. Optimised flanking primers creating a ∼120 bp PCR product for HRM (20 bp each, Tm 60°C) and a 200-400 bp product for DNA sequencing were selected for each gRNA with Primer 3 software (Koressaar and Remm, 2007) and oligos bought from MWG Eurofins. CRISPR oligos were annealed and ligated into BsaI-digested pDR274 (Addgene plasmid #42250), the plasmid DNA was purified, sequenced and digested with DraI, and the 284 bp fragment was gel purified and used to synthesise gRNA with T7 RiboMAX large-scale RNA production kit (Promega). gRNA was phenol/chloroform purified, ethanol precipitated, quantified by gel and Qubit (Invitrogen), aliquoted in 5 µl samples at 2 µg/µl and stored at −80°C. NotI-HF-linearised pCS2-Cas9 was transcribed using mMessage mMachine SP6 kit (Ambion) and product purified as for gRNA.

HRM-selected AB wild-type fish were DNA sequenced over the target loci to avoid polymorphisms, crossed and the resulting embryos injected with 1 nl containing 150 pg gRNA, 200 pg Cas9 mRNA or 200 pg of each TALEN with 0.03% rhodamine dextran to select injected embryos. Ten 48 hpf larvae were analysed by HRM to verify mutagenesis, their F0 siblings grown to adulthood and outcross F1 progeny analysed for transmission by HRM. Mutant loci of F1 heterozygotes were sequenced to identify F0s transmitting mutations of interest and F1 siblings that had grown to adulthood, and F1 heterozygotes were identified by HRM and sequencing of fin-clip DNA. Subsequent generations were bred by outcross to wild-type AB selected as non-polymorphic at the target locus. To generate double mutants, *larp6a^kg139/kg139^* and *larp6b^+/kg153^* F2 heterozygotes were crossed to obtain F3 progeny in which 50% of fish were dual heterozygotes (*larp6a*^+/^*^kg139^;larp6b*^+/*kg153*^), which were then in-crossed to generate F4 progeny of which 6.25% were predicted to be homozygous for both genes (*larp6a^kg139^*^/*kg139*^*;larp6b^kg153^*^/*kg153*^). F4 individuals were in-crossed to generate MZ mutants at F5.

### Imaging, *in situ* mRNA hybridization and immunodetection

*In situ* mRNA hybridization and immunodetection were performed as described previously (Hinits et al., 2007). Digoxigenin-labelled probes for *larp6a, squint* and *chordin* were synthesised from linearised plasmid vectors pCS2+larp6a (Wang et al., 2009; a generous gift from R. Karlstrom, University of Massachusetts, USA) and pBluescriptSK-squint (a kind gift from C. Houart, King's College London, UK). A probe against *larp6b* was made by PCR either from the BC134173.1 template or from 1 dpf cDNA using listed primer pairs (Table S4) with an added T7 polymerase-binding site. For *in situ* imaging, embryos were immersed in glycerol and images collected on a Leica MZ16F with LED light attachment, Olympus DP70 camera and DP Controller. Primary antibody F59 (1:5), against slow myosin was detected with goat anti-mouse IgG1-Alexa 555 (Serotec). For confocal imaging, embryos were mounted in glycerol, Citifluor (Agar) or 0.8-1% low melting point agarose and data were collected using a LSM Exciter microscope (Zeiss) equipped with 20×/1.0 W objective and subsequently analysed using Fiji (www.Fiji.sc) or ZEN (Zeiss) software.

### Adult fish analysis

Siblings (5 mpf) from single heterozygote in-crosses were anaesthetised with tricaine (Sigma-Aldrich), sexed, blotted dry and weighed using an Ohaus YA102 balance. Standard length was measured against a ruler and fish were fin-clipped for sequence genotyping. Weights and lengths were compared using ANOVA.

### Chorion diameter size

Mean chorion diameter for each clutch was determined by measuring the maximal chorion width of a series of eggs in a single orientation in an image of 17-80 embryos/clutch using Fuji. Yolk size was measured as maximal width of yolk orthogonal to the animal-vegetal axis. Size-corrected chorion expansion was calculated as (chorion diameter×100)/yolk diameter. Clutch mean was used for further comparisons.

### Egg activation timelapse

Eggs squeezed from females of the indicated genotype were maintained in Hank's medium (0.137 M NaCl, 5.4 mM KCl, 0.25 mM Na_2_HPO_4_, 0.44 mM KH_2_PO_4_, 1.3 mM CaCl_2_, 1 mM MgSO_4_ and 4.2 mM NaHCO_3_) and placed in a glass depression slide on a Zeiss Axiophot with 20× NA0.5 Plan-Neofluar objective under DIC. Medium was diluted with distilled water and t=0 s image collected within 15 s and serially thereafter on an Olympus DP70 camera.

### Transmission electron microscopy

Eggs were squeezed from gravid females as described previously (Westerfield, 2000), stored in Hank's medium and fixed overnight at 4°C in 2.5% (v/v) glutaraldehyde with 0.1 M PIPES buffer (pH 7.2) complemented with Hank's salts. After squeezing, females (two wild types and one double mutant) were killed using excess anaesthetic, whole ovaries were dissected as described (Elkouby and Mullins, 2017) and fixed as above. The next day, samples were washed briefly with 0.1 M PIPES buffer (pH 7.2) and post-fixed in 1% (v/v) osmium tetroxide in water. Samples were then dehydrated through a graded ethanol series before infiltration with Spurr resin (TAAB) and polymerization at 60°C for 24 h. Semi-thin sections were cut at ∼450 nm and stained with 1% Toluidine Blue. Ultrathin sections (80 nm) were cut using an ultramicrotome (UC 7, Leica Microsystems), mounted on grids and post-stained with Uranyless stain (TAAB S474) and Reynolds lead citrate (Reynolds, 1963). Samples were examined using a TEM operated at 120 Kv (JEOL JEM 1400Plus, JEOL, Japan). Images were acquired with a 2k by 2k format CCD camera (JEOL Ruby CCD Camera). At least ten squeezed eggs and five oocytes at each developmental stage (identified according to Selman et al., 1993) were examined in each sample and the phenotype in wild-type or mutant samples matched the results shown in all cases.

### Protein extraction

Chorions were dissected manually from 3- to 4 h-old fertilised lays and 30 chorions washed into 40 μl of TNE buffer [100 mM Tris-HCl (pH 6.8), 100 mM NaCl, 10 mM EDTA, 0.5% Tween20] followed by extraction in 10 μl of SDS PAGE sample buffer [250 mM Tris-HCl (pH 6.8), 10% SDS, 30% glycerol, 10 mM DTT and 0.05% Bromophenol Blue] at 95°C for 15 min. Samples equivalent to the extraction of 15 chorions (25 μl) and PageRuler Plus Prestained Protein Ladder (Thermo Fisher Scientific) were run on 4-20% acrylamide gels (Bio-Rad) at 185 V, stained in Coomassie Brilliant Blue, photographed on GelDoc XR+ and analysed in ImageJ.

### Sample preparation and LC-MS/MS analysis

Each chorion replicate protein sample (prepared as above without Bromophenol Blue from 30 chorions, containing 260-300 µg) was reduced with 100 mM DTT at 95°C for 10 min, before being subjected to trypsin digestion using Filter Aided Sample Preparation (FASP) protocol with some modifications (Wiśniewski et al., 2011). Briefly, reduced samples were diluted 1 in 7 in UA buffer [8 M urea, 100 mM Tris-HCl (pH 8.5)], transferred to Vivacon-500 30 kDa centrifugal filter units (Sartorius) and concentrated by centrifugation at 14,000 ***g*** for 15 min. Filters were then washed twice by addition of 0.2 ml of UA buffer and re-concentrating by centrifugation as before. Subsequently, the proteins were alkylated by addition of 100 µl of 50 mM iodoacetamide in UA buffer and incubation at room temperature in the dark for 30 min. The iodoacetamide solution was then removed by centrifugation at 14,000 ***g*** for 10 min, and samples were washed twice with 0.2 ml of UA buffer as before. This was followed by three washes with 0.2 ml of ABC buffer (0.04 M ammonium bicarbonate in water) prior to transferring the filters to new collection tubes, and addition of digestion buffer [0.3 µg of MS grade Trypsin (Sigma-Aldrich) in 50 µl of ABC buffer per filter]. The proteins were digested overnight in a thermo-mixer at 37°C with gentle shaking (600 rpm). The next day, the resulting peptides were eluted from the filters by centrifugation at 14,000 ***g*** for 10 min, followed by two further elutions, each with 100 µl of the ABC solution. The combined eluates were then reconstituted in 2% acetonitrile (ACN), 0.2% trifluoroacetic acid (TFA) and desalted using C18 StageTips (Rappsilber et al., 2007). The peptides were then dried in a speedvac and re-suspended in A* buffer (2% ACN, 0.5% acetic acid and 0.1% TFA in water) before LC-MS/MS analysis. Approximately 1 µg of each digested sample was analysed on a Q-Exactive plus Orbitrap mass spectrometer coupled with a nanoflow ultimate 3000 RSL nano HPLC platform (Thermo Fisher Scientific). Briefly, samples were resolved at a flow rate of 250 nl/min on an Easy-Spray 50 cm×75 μm RSLC C18 column with 2 µm particle size (Thermo Fisher Scientific), using a 123 min gradient of 3% to 35% of buffer B (0.1% formic acid in ACN) against buffer A (0.1% formic acid in water), and the separated peptides were infused into the mass spectrometer by electrospray. The spray voltage was set at 1.95 kV and the capillary temperature was set to 255°C. The mass spectrometer was operated in data-dependent positive mode, with 1 MS scan followed by 15 MS/MS scans (top 15 method). The scans were acquired in the mass analyser at the 375-1500 m/z range, with a resolution of 70,000 for the MS and 17,500 for the MS/MS scans. Fragmented peaks were dynamically excluded for 30 s. Three biological replicates per each experimental group (wild type, *larp6a^−/−^*, *larp6b^−/−^* and *larp6a^−/−^;larp6b^−/−^*) were analysed.

### Proteomics data analysis

MaxQuant (version 1.6.3.3) software was used for database search and label-free quantification of mass spectrometry raw files (Tyanova et al., 2016a). The search was performed against a FASTA file of the *Danio rerio* proteome, extracted from uniprot.org. A precursor mass tolerance of 4.5 ppm, and a fragment mass tolerance of 20 ppm was applied. Methionine oxidation and N-terminal acetylation were included as variable modifications while carbamidomethylation was applied as a fixed modification. Two trypsin mis-cleavages were allowed, and the minimum peptide length was set to seven amino acids. Default MaxQuant parameters for Label-Free Quantification (LFQ) were used. All raw files were searched together, with the match between runs option enabled. All downstream data analysis was performed using Perseus (version 1.5.5.3) (Tyanova et al., 2016b), using the MaxQuant ProteinGroups.txt output file. Briefly, protein LFQ intensities were converted to a Log 2 scale. Reverse (decoy) hits, potential contaminants and proteins identified only by modified peptides were filtered out. Proteins with less than three valid values in at least one experimental group were also filtered out. Missing intensity values were then imputated for each replicate run, using a normal distribution with a width of 0.3 and a downshift of two standard deviations. The comparison of wild type with each knockout group was then carried out using a two-sided two-sample *t*-test analysis, with an S0 of 0.1 and a permutation based False Detection Rate (FDR) of 5%, derived from 500 randomisations. Hierarchical clustering was performed in Perseus using average Euclidean distances. All mass spectrometry raw files and search results reported here have been deposited at the ProteomeXchange Consortium via the PRIDE database (www.ebi.ac.uk/pride/) (Vizcaíno et al., 2014) under accession number PXD017123.

### Statistical analyses

Statistical analyses used Excel, SPSS or Statplus:mac v5 for ANOVA to assess significant differences between mutant and sibling groups with Bonferroni post-hoc tests. χ^2^ test on Excel was used to analyse differences between distributions. Unless otherwise stated, data are expressed as mean±s.e.m. with numbers on columns representing the number of fish analysed. *P*-values are indicated for each experiment.

## Supplementary Material

Supplementary information
