# Supplementary material for: Maternal Larp6 controls oocyte development, chorion formation and elevation
Source: Development. 2020 Feb 26;147(4):dev187385. doi: 10.1242/dev.187385 (PMC7055395; doi:10.1242/dev.187385)
Supplement: Supplementary information [file develop-147-187385-s1.pdf]

Table S1 Mendelian frequencies of offspring of each genotype in crosses of *larp6a* and *larp6b*.

Gen	Maternal genotype	Paternal genotype	Offspring genotype	Number of offspring	Proportion (%)	Difference to expected ratio? X^2 test (P)
F2	*larp6a*$^{+/-}$	*larp6a*$^{+/-}$	*larp6a*$^{+/+}$	12	25	0.779
			larp6a$^{+/-}$	26	54	
			larp6a$^{-/-}$	10	21	
F2	*larp6b*$^{+/-}$	*larp6b*$^{+/+}$	*larp6b*$^{+/+}$	17	50	1.000
			larp6b$^{+/-}$	17	50	
F2	*larp6b*$^{+/-}$	*larp6a*$^{-/-}$	*larp6a*$^{+/-}$;*larp6b*$^{+/+}$	26	51	0.889
			larp6a$^{+/-}$;*larp6b*$^{+/-}$	25	49	
F3	*larp6a*$^{+/-}$	*larp6a*$^{+/-}$	*larp6a*$^{+/+}$	15	29	0.772
			larp6a$^{+/-}$	24	46	
			larp6a$^{-/-}$	13	25	
F3	*larp6b*$^{+/-}$	*larp6b*$^{+/-}$	*larp6b*$^{+/+}$	13	21	0.760
			larp6b$^{+/-}$	33	53	
			larp6b$^{-/-}$	16	26	
F3	*larp6a*$^{+/-}$;*larp6b*$^{+/-}$	*larp6a*$^{+/-}$;*larp6b*$^{+/-}$	*larp6a*$^{+/+}$;*larp6b*$^{+/+}$	7	11	0.740
			larp6a$^{+/+}$;*larp6b*$^{+/-}$	7	11	
			larp6a$^{+/+}$;*larp6b*$^{-/-}$	4	7	
			larp6a$^{+/-}$;*larp6b*$^{+/+}$	5	8	
			larp6a$^{+/-}$;*larp6b*$^{+/-}$	15	25	
			larp6a$^{+/-}$;*larp6b*$^{-/-}$	9	15	
			larp6a$^{-/-}$;*larp6b*$^{+/+}$	4	7	
			larp6a$^{-/-}$;*larp6b*$^{+/-}$	5	8	
			larp6a$^{-/-}$;*arp6b*$^{-/-}$	5	8	
F4	*larp6a*$^{-/-}$;*larp6b*$^{-/-}$	*larp6a*$^{-/-}$;*larp6b*$^{-/-}$	*larp6a*$^{-/-}$;*larp6b*$^{-/-}$	24	100	1.000
	larp6a$^{-/-}$;*larp6b*$^{-/-}$	*larp6a*$^{-/-}$;*larp6b*$^{-/-}$	*larp6a*$^{-/-}$;*larp6b*$^{-/-}$	6	100	1.000

Note that only the embryos molecularly genotyped are reported; lays were much larger.

Table S2 MS/MS analysis of chorion

See online Excel file. The sheet named Key provides an indication of the colour code and values in the data pages.

Click here to Download Table S2

Development • Supplementary information

Table S3 Primers used in *larp6* mutagenesis and analysis

Gene	Target site	Forward oligo	Reverse oligo	Vector	WT fragment size
larp6a exon 1	GGAGGACGATGAACCGGACG	5'-TA*GGAGGACGATGAACCGGACG*-3'	3'-*TCCTGCTACTTGGCCTGC*CAAA-5'	pDR274	n/a
larp6a	HRM primers	5'-AGATCAGCGCTCCGGTCA-3'	5'-CGGTCTGGAGCTCGATGG-3'	n/a	102 bp
larp6a	Sequencing primers	5'-CTGGGGAGTTTGTGTTTTCC-3'	5'-CCAGCATTATTCACCCCAGT-3'	n/a	317 bp
larp6b	HRM primers	5'-TGTCCTCACCCGGTTTTACTG-3'	5'-TCCGTATCTTACCCATCC-3'	n/a	115 bp
larp6b	Sequencing primers	5'- TGCACGTGGAAGCAGACAG-3'	5'-CTTCAACAACAAACACAACGTCT-3'	n/a	280 bp
	**Left TALEN binding site**	**Target site**	**Right TALEN binding site**		
larp6b exon 1	3'-AGAAGGTAACACTTCTGT-5'	5'-CCCAACAA*T*CAAAGGG-3'	5'-TCTGTTCTCATACACTGA-3'		n/a

Table S4 Primers for ISH probe synthesis,RT-PCR, PCR for RNA splicing

Probe	Forward primer	Reverse primer
larp6b ISH probe	5'- TGTCCTCACCCGGTTTTACT-3'	5'-GCACTGCACAAAGGCTCATA-3'
larp6a RT PCR	5'-ATCTCAGTGACACCGTAT-3'	5'- AAGGACAACAGAAATCAAATC-3'
larrp6b RT PCR	5'- TTACTGACATCTTCCATTG-3'	5'- AACTCCCATCCAAACTAT-3'
larp6a UTR (splicing test)	5'- GGGAGTTTGTGTTTTCCGCT-3'	5'- ACTCTTTCACTGTGCGGAGA-3'
larp6b UTR (splicing test)	5'- AGAATCACCTGTGCCCGG-3'	5'- GCACAAAGGCTCATACCATTTC-3'

Development • Supplementary information

Fig. S1

A

```
D. rerio Larp6a ENSDARP00000019822/1-471
D. rerio Larp6b ENSDARP00000153929/1-485
Human LARP6

Larp6b MSSPGF------TDIFHC----------EDTQQSKGL---------------FSYTDDGLNDSL
Larp6a MSSE—QPPREI--SAPVTITVAIQAAEEDDEPDEEPSCNTIELQT--------GSGSEDELGRH
LARP6  MAQSGGEARPGPKTA-VQIRVAIQEAEDVDELEDEEEGAETRGAGDPARYLSPGWGSASEEEPS
       *..                            .*.::::

Larp6b ---DGSSTDLTDVFEEDFCEGELWIPPNDDVTQQIATQLENYLSDENLSDDAFLLKHVQR
Larp6a DKSSGAGTSGGEL-EE-----ESWQPPDPELIQKLVAQIEYYLSDENLEHDAFLLKHVRR
LARP6  RGHSGT—TASGGENEREDLEQE-WKPPDEELIKKLVDQIEFYFSDENLEKDAFLLKHVRR
          .*: *  :.  *    * * * **: :: :::. *:* *.*****..********:*

Larp6b NKMGYVSLKLLTSFKKIRDLTRDWRTTLAAARTSLQLEVNEMGTKVRRRTPVPDWLLCIP
Larp6a NKLGFVSVKLLTSFKKVKHLTRDWRTTAYALRHSNLLELNDEGRKVRRRSTVPVFASESL
LARP6  NKLGYVSVKLLTSFKKVKHLTRDWRTTAHALKYSVVLELNEDHRKVRRTTPVPLFPNENL
       **:*:**:********::.********  * : *  **:*:    ****.: ** :    .

Larp6b TSKLLLAWNFLDGAGPVK-EKFESPGVE------QLGIMEAAMRVFSPYGTISSLRILRP
Larp6a PSRMLLLSELKRWPELGIALGGD—-SNNGSGPTQQERLMELLLKAFGNYGPIASVRVLKP
LARP6  PSKMLLVYDLYLSPKL-WALATP-----QKNGRVQEKVMEHLLKLFGTFGVISSVRILKP
       .*::**  ::                  .     *..:** .::.*. :* *:*:*:::*

Larp6b GKEIPAELKRYTKKHLELGRKVCAVVEYEYLEGARKAFEALKVEEQ-QGGRGICVVLLGS
Larp6a GKDLPADLKKLSGRYSQLGTEECAIVEFEEVEAAMKAHEAVGGEGGNRGPLGLKVVLIGT
LARP6  GRELPPDIRRISSRYSQVGTQECAIVEFEEVEAAIKAHEFMITES--QGKENMKAVLIGM
       *:::* :::: : :: ::* : **:**:* :*.* **.* :   *    :*   .: .**:*

Larp6b RGTRKPGCSQGLVDEEQEDCIDIDVLKRPDRKARQFVYSLEDSAVCSSSESDFAPASPRP
Larp6a KPPKKKVPKD-RPRDEGIGGM--RKSRSLNSRVRELQYHGDD-SAASSSETESNPTSPRL
LARP6  KPPKKKPAKDKNHDEEPT--ASIHLNKSLNKRVEELQYMGDESSANSSSDPESNPTSPMA
       :..:*.  .:    **     .:  :   : :..:: *  ::*:. ***: :  *:**

Larp6a NRRVSRPQALYGSPLAIPRVSTFR-SDPYRNPLGSPVGSPLQ----PRKLFPCSHVTSPL
Larp6b ARKSRSCNKLSPTSAG------PNHLSPVVSPRSSPWSSPRASPCTQRKT-H-PSGKSPL
LARP6  GRRHAATNKLSPSGHQNLFLS-PNA-SPCTSPWSSPLAQ----RK-----GVS--RKSPL
        *:    : *  :        :     : * :.*: ** ..          .   .***

Larp6b ATHP---FSSTPSGAGTSFKYKASGELSPDDLGFTSSPWVQRRKNAAQVIQP-EMASS--
Larp6a ASEGRL--SPEPG—-RRWADYSS-----DSSLTPSGSPWVQRRKQ------VASQESSPV
LARP6  AEEGRLNCSTSPEIFRKCMDYSS-----DSSVTPSGSPWVRRRRQAEMGTQEKSPGTSPL
       *        *  *        *      ..:    ::.****:**::.          **

Larp6b MSPSQLIKSLS-----VLVVRQPVGPDGTKGFHNCIGRGKVLLPQ
Larp6a GSPMLARKIQNADGLPPGVVRLPRGPDGTRGFHCP-PLGKTASTQTCL
LARP6  ----LSRKMQTADGLPVGVLRLPRGPDNTRGFHGH-ERSRA-----CV
           *    .          *:* * ***.*:***       .:.
```

KEY

La Module, formed by La-motif (composed of LaM and RRM1).

Nuclear Export Signal = dark grey – overlap with RRM1 (Merret at al 2013).

Nuclear Localisation Signal = orange – overlap with RRM1 (Merret at al 2013).

Yellow is well-conserved but of no known function.

Suz-C human = 453-482 – overlaps LSA, some similarity with zebrafish Larp6a, but little with Larp6b (Song et al, 2008).

LSA human = 468-488 – highly conserved in both Larp6a and Larp6b (Bousquet-Antonelli & Deragon, 2009).

B

LARP6 paralogues

Common name (phylogenetic position)	Species Name	Protein name	BfLARP6	DmLARP6	DrLARP6a	DrLARP6b	GgLARP6a	GgLARP6b	HsLARP6	IpLarp6a	Iplarp6b	LcLarp6	LgLARP6	LoLarp6	MdLAR6a	MdLAR6b	MmLARP6	NvLARP6	OcLARP6	OlLarp6a	OlLarp6b	OtLARP6	RnLARP6	StpLARP6	TaLARP6	TgLARP6a	TgLARP6b	VcLARP6	XtLARP6a	XtLARP6b
Amphioxus (basal chordate)	Branchiostoma floridae	BfLARP6	100																											
Fly (protostome)	Drosophila melanogaster	DmLARP6	29	100																										
Zebrafish (clupeomorph fish)	Danio rerio	DrLARP6a	38	33	100																									
Zebrafish (clupeomorph fish)	Danio rerio	DrLARP6b	34	27	39	100																								
Chicken (bird)	Gallus gallus	GgLARP6a	36	31	61	36	100																							
Chicken (bird)	Gallus gallus	GgLARP6b	33	31	37	39	34	100																						
Human (mammal)	Homo sapiens	HsLARP6	38	33	60	35	70	36	100																					
Channel catfish (clupeomorph fish)	Ictalurus punctatus	IpLarp6a	35	31	83	38	59	36	60	100																				
Channel catfish (clupeomorph fish)	Ictalurus punctatus	Iplarp6b	32	26	37	66	35	36	35	38	100																			
Coelacanth (basal lobe-finned fish)	Latimeria chalumnae	LcLarp6	39	31	65	39	68	36	66	60	37	100																		
Limpet (protostome)	Lottia gigantae	LgLARP6	46	33	41	35	40	37	40	41	34	41	100																	
Spotted gar fish (basal ray-finned fish)	Lepisosteus oculatus	LoLarp6	36	30	76	38	61	35	63	72	37	62	41	100																
Opposum (marsupial)	Monodelphis domestica	MdLAR6a	38	32	57	36	68	37	74	57	35	64	40	59	100															
Opposum (marsupial)	Monodelphis domestica	MdLAR6b	28	31	38	38	34	47	36	36	36	37	36	38	35	100														
Mouse (mammal)	Mus musculus	MmLARP6	38	33	60	35	70	36	91	61	34	66	40	63	74	35	100													
Sea anemone (diploblast)	Nematostella vectinis	NvLARP6	39	30	36	34	37	35	36	36	35	36	43	35	37	34	35	100												
Rabbit (mammal)	Oryctolagus cuniculus	OcLARP6	38	30	58	34	65	38	80	58	34	62	39	61	67	37	79	35	100											
Medaka fish (euteleost)	Oryzias latipes	OlLarp6a	37	30	81	38	60	37	60	73	37	60	42	70	57	37	60	37	58	100										
Medaka fish (euteleost)	Oryzias latipes	OlLarp6b	27	21	31	36	30	32	30	30	34	28	29	29	29	34	30	28	30	30	100									
Unicellular marine alga	Ostreococcus tauri	OtLARP6	25	26	28	27	25	26	26	28	25	26	30	28	25	29	26	24	25	28	21	100								
Rat (mammal)	Rattus norvegicus	RnLARP6	37	33	60	35	70	35	90	61	35	66	40	63	74	35	96	36	78	61	29	25	100							
Sea urchin (deuterostome)	Strongylocentrotus purpurat	StpLARP6	45	33	38	32	38	32	38	36	31	39	43	39	37	32	38	36	36	38	25	27	38	100						
Trichoplax (basal metazoan)	Trichoplax adhaerens	TaLARP6	37	28	32	31	32	32	35	32	29	35	39	33	35	32	35	36	34	32	26	22	35	35	100					
Zebrafinch (bird)	Taeniopygia guttata	TgLARP6a	37	31	61	36	86	34	68	60	34	66	40	60	66	35	68	37	63	59	30	25	67	38	33	100				
Zebrafinch (bird)	Taeniopygia guttata	TgLARP6b	31	30	39	37	36	59	38	39	36	37	34	37	35	46	38	32	40	39	34	28	38	33	29	34	100			
Volvox (unicellular)	Volvox carteri	VcLARP6	32	27	30	25	29	30	30	29	24	28	34	31	30	28	29	31	30	28	21	26	30	30	28	29	28	100		
Frog (tetrapod)	Xenopus tropicalis	XtLARP6a	39	32	64	39	70	37	69	63	37	71	41	64	68	36	68	37	62	63	29	28	68	41	33	69	37	30	100	
Frog (tetrapod)	Xenopus tropicalis	XtLARP6b	36	29	41	45	40	46	40	40	42	42	40	41	40	40	40	35	40	41	31	26	41	34	32	40	44	28	41	100

Figure S1 Larp6 genes in zebrafish

A. Protein alignment of Larp6a and Larp6b with human LARP6 highlighting the major conserved domain structure diagnostic of Larp6 family.

B. Table showing the percentage amino acid identity of various Larp6s. Alignment was made using T-Coffee on the dataset from Merret et al (2013) plus the additional proteins shown. Colours highlight evolutionary relationships. Note that the metazoan Larp6a and Larp6b gene duplication preceded the vertebrate radiation and Larp6b was secondarily lost in mammals.

Development • Supplementary information

Fig. S2

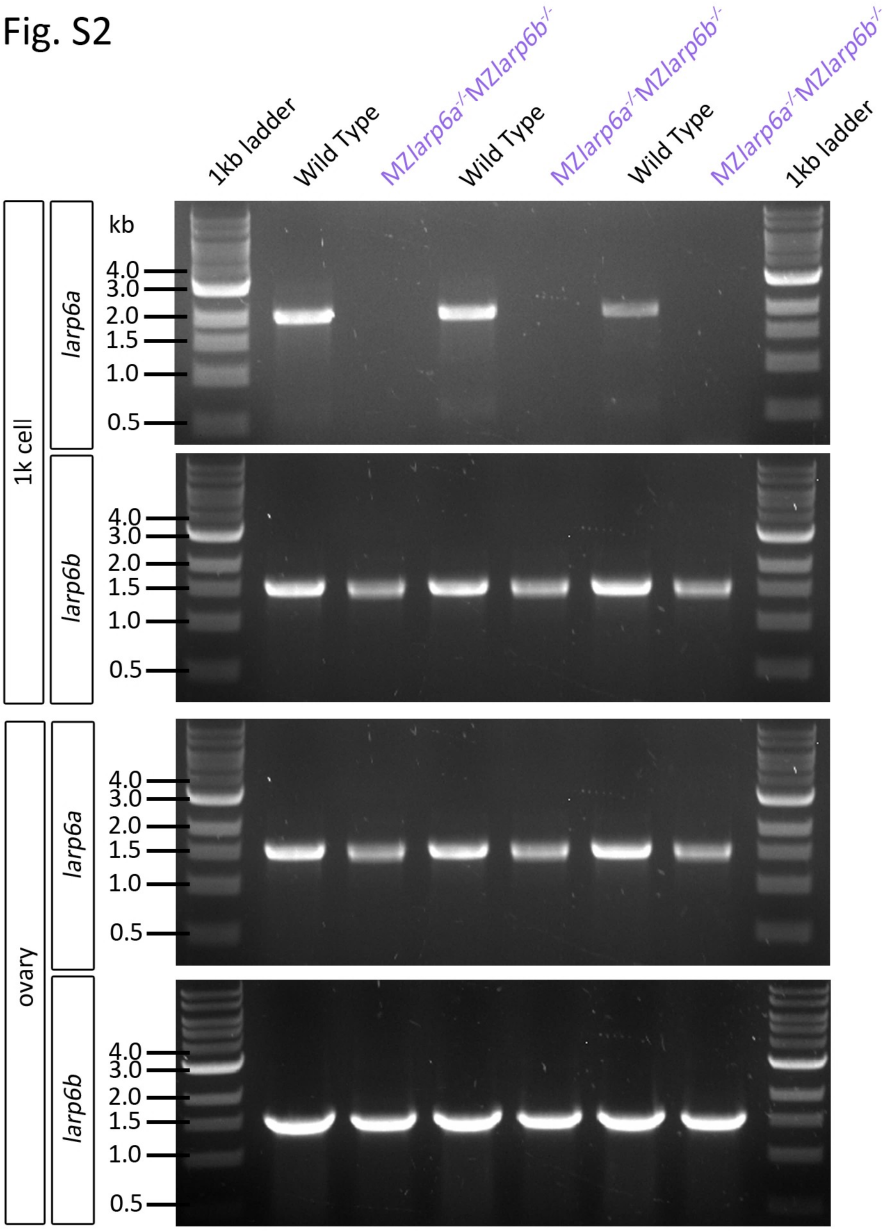

Figure S2 Lack of alternative mRNA transcripts in Larp6 mutant embryos and ovaries RNA isolated from 1K cell stage embryos derived from three replicate *MZlarp6a$^{-/-}$;MZlarp6b$^{-/-}$* double mutant incrosses, or dissected ovaries from three separate adult *MZlarp6a$^{-/-}$;MZlarp6b$^{-/-}$* females was reverse transcribed and cDNA subjected to PCR for *larp6a* and *larp6b*. No aberrant transcripts were detected in wild type or mutant embryos or ovaries, despite consistent reduction in wild type transcript in mutant tissues which was marked for *larp6a* mRNA and significant for *larp6b* mRNA.

Fig. S3

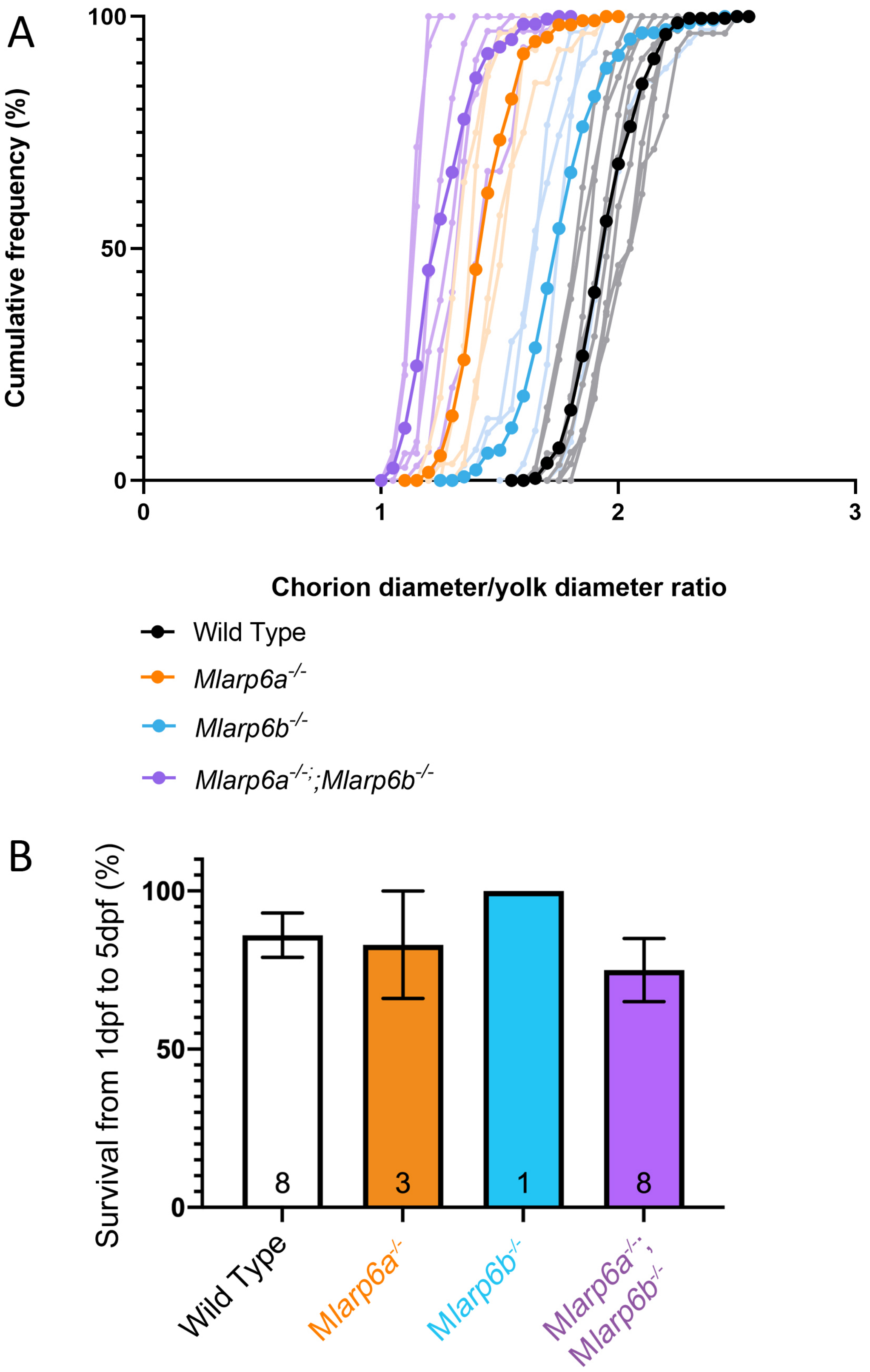

Figure S3 MZ mutants show reduced chorion diameter relative to yolk diameter but good survival beyond 1 dpf.

A. Reduction of chorion diameter in mutant lays irrrespective of the overall size of eggs in a clutch.

B. Survival of mutant clutches did not differ from wild type beyond 28 hpf. Samples are a subset of lays from Fig. 4D.

Development • Supplementary information

Fig. S4

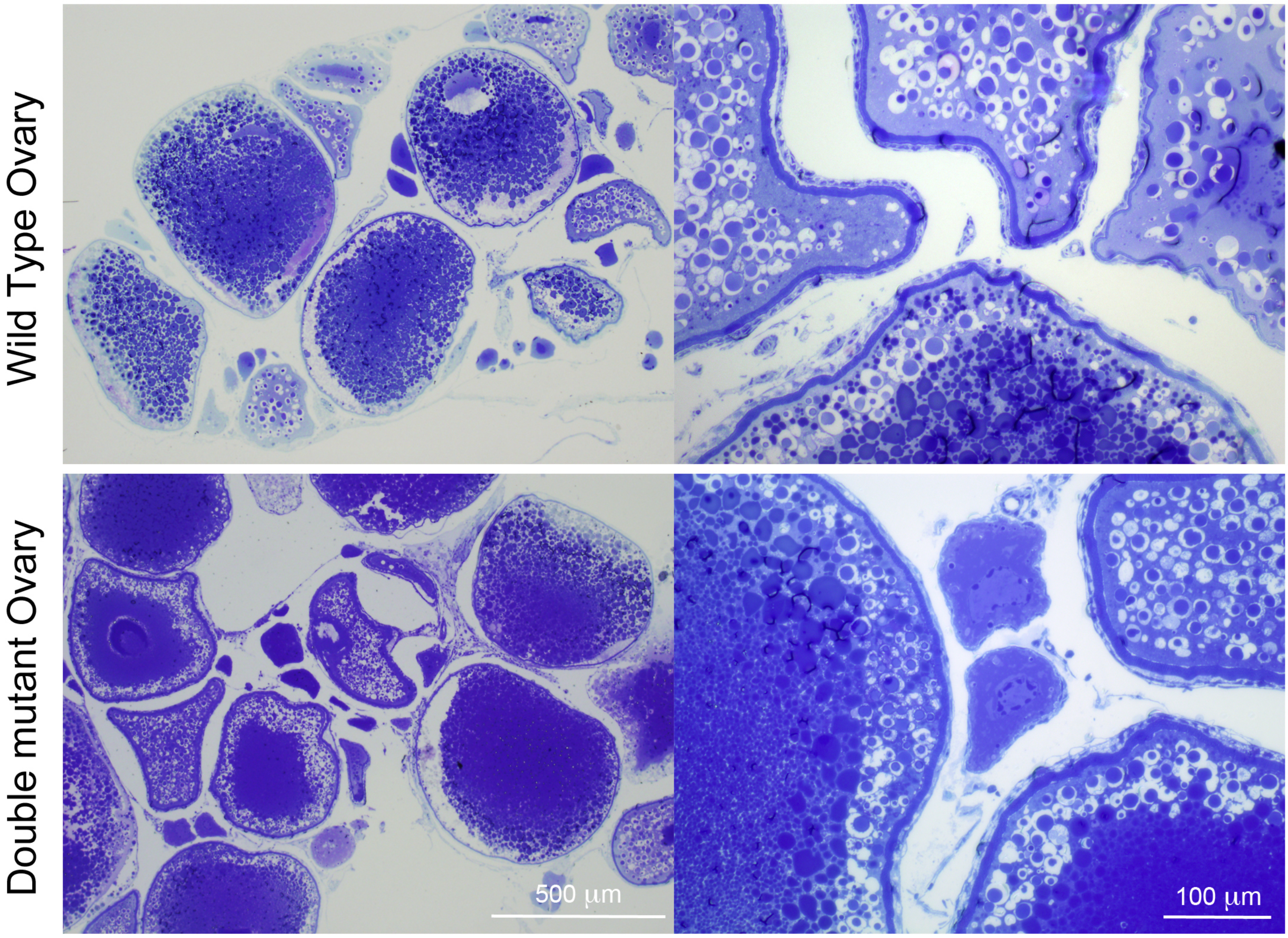

Figure S4 Histology of ovaries from wild type and *larp6a$^{-/-}$;larp6b$^{-/-}$* double mutant females. Semithin sections of ovaries reveal a similar range of oocyte sizes and stages between wild type and mutant. Even higher magnification light microscopy (right) fails to reveal obvious differences between mutant and wild type oocytes or follicular cells. Somewhat stronger Toluidine Blue staining in the mutant sections may reflect minor differences in processing of the blocks.

Fig. S5

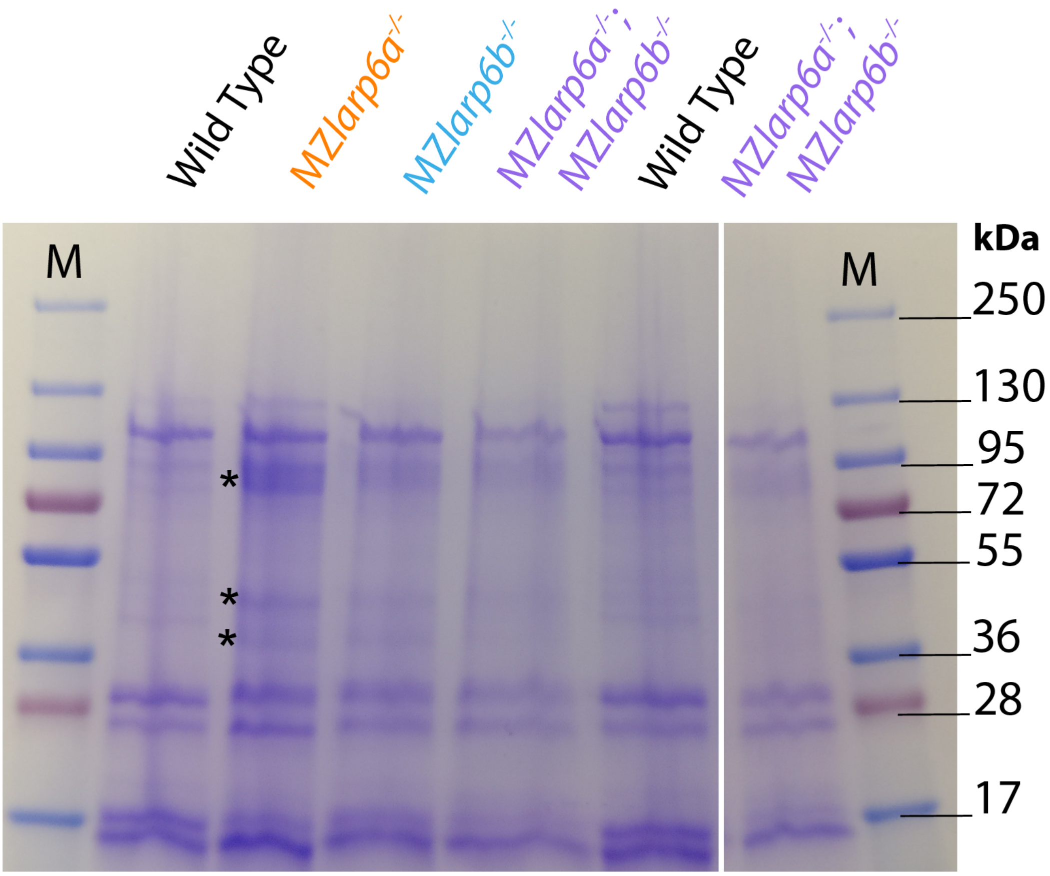

Figure S5 Altered chorion proteins in maternal mutant embryos.
SDS PAGE of chorion proteins extracted from six separate 3-4 hpf lays from the indicated mutant incrosses and AB (Wild Type) incrosses show consistent differences between mutant and wild type, with more Coomassie Blue stain in the 90 kDa and 40-50 kDa regions (asterisks). Note the greater intensity of Coomassie stained bands from *MZlarp6a$^{-/-}$* and lower from *MZlarp6a$^{-/-}$; MZlarp6b$^{-/-}$*, compared to Wild Type and *MZlarp6b$^{-/-}$*, which were consistent findings. Two irrelevant lanes were cut from the gel image. M markers.
